# Transcriptome Sequences Resolve Deep Relationships of the Grape Family

**DOI:** 10.1371/journal.pone.0074394

**Published:** 2013-09-17

**Authors:** Jun Wen, Zhiqiang Xiong, Ze-Long Nie, Likai Mao, Yabing Zhu, Xian-Zhao Kan, Stefanie M. Ickert-Bond, Jean Gerrath, Elizabeth A. Zimmer, Xiao-Dong Fang

**Affiliations:** 1 Department of Botany, National Museum of Natural History, MRC166, Smithsonian Institution, Washington, D.C., United States of America; 2 BGI-Shenzhen, Shenzhen, China; 3 Key Laboratory of Biodiversity and Biogeography, Kunming Institute of Botany, Chinese Academy of Sciences, Kunming, Yunnan, China; 4 College of Life Sciences, Anhui Normal University, Wuhu, Anhui, China; 5 UA Museum of the North Herbarium and Department of Biology and Wildlife, University of Alaska Fairbanks, Fairbanks, Alaska, United States of America; 6 Department of Biology, University of Northern Iowa, Cedar Falls, Iowa, United States of America; Universidad Miguel Hernández de Elche, Spain

## Abstract

Previous phylogenetic studies of the grape family (Vitaceae) yielded poorly resolved deep relationships, thus impeding our understanding of the evolution of the family. Next-generation sequencing now offers access to protein coding sequences very easily, quickly and cost-effectively. To improve upon earlier work, we extracted 417 orthologous single-copy nuclear genes from the transcriptomes of 15 species of the Vitaceae, covering its phylogenetic diversity. The resulting transcriptome phylogeny provides robust support for the deep relationships, showing the phylogenetic utility of transcriptome data for plants over a time scale at least since the mid-Cretaceous. The pros and cons of transcriptome data for phylogenetic inference in plants are also evaluated.

## Introduction

The grape family (Vitaceae) has been widely recognized for its economic importance as the source of table grapes, wine, and raisins. The family consists of 14 genera and ~ 900 species [1]. Many species of the family are dominant lianas in lowland tropical forests, while species in *Parthenocissus* Planchon, *Ampelopsis* Michx. and *Vitis* L. are primarily from the temperate zone. Previous phylogenetic analyses support five major clades within Vitaceae: (i) the *Vitis* – *Ampelocissus* –clade (180 spp.), (ii) the *Ampelopsis* – *Rhoicissus* clade (43 spp.), (iii) the *Parthenocissus* -*Yua* clade (15 spp.), (iv) the core *Cissus* clade (300 spp.), and (v) the *Cayratia* – *Tetrastigma* – *Cyphostemma* – clade (350 spp.) [2,3]. *Parthenocissus* and *Yua* are supported as closely related to *Vitis* (3, 4). However, in spite of several recent efforts [2,3,5,6,7,8] that effectively resolved the relationships within each of the main clades, the deep relationships of the family remained poorly resolved. Recently, it has been demonstrated for a number of plant and animal lineages that uncertainty of deep relationships among taxonomic groups hinders progress in understanding their evolution including their temporal and spatial origins as well as their morphological changes over time [9,10]. In particular, biogeographic reconstructions, especially at the family level, are a major challenge for plant biologists [2,3,11–16], even though methods have been developed to account for phylogenetic uncertainty in biogeographic inferences [17–19].

Transcriptome sequences, generated using high throughput techniques, have been shown to provide a rich set of characters to produce phylogenies in eukaryotes and are more efficient and cost-effective than traditional PCR-based and EST-based methods (20). Recent studies have demonstrated the utility of transcriptome data for resolving the relationships of mosquitoes [20], mollusks [9,21], and the large tetrapod group consisting of turtles, birds and crocodiles [22]. For example, even though mollusks have an excellent fossil record, deep relationships of the phyllum have been uncertain when molecular phylogenies used a few genes. With a transcriptome approach, the major clades were resolved with highly significant statistical support. Given its potential, we decided to take a phylogenomics approach to resolve the deep relationships of the Vitaceae. This represents the first study in plants to use RNA-Seq data to reconstruct phylogenies in flowering plants. Several previous studies employed RNA-Seq data to explore the evolution of paleopolyploidy (e.g. [23-25]; also see 26). The 1KP collaborative project has also generated large-scale gene sequence information for many different species of plants (http://www.onekp.com/).

## Results and Discussion

### Backbone relationships of the grape family

Transcriptome (RNA-Seq) data were obtained from 14 species of the grape family and one species of its sister family Leeaceae (Table S1, Figure S1), and augmented with publicly available whole genome data of the domesticated grape *Vitis vinifera* [27]. Each of the five major lineages of the grape family [3] was represented in the data. We obtained about twenty million 90 bp paired-end DNA sequence reads from non-normalized cDNA libraries for each of the 15 species using an Illumina HiSeq 2000, assembled the sequence reads *de novo* and retained all contigs ≥ 150 bp for further analysis (Table S2). This strategy identified 417 orthologous genes suitable for concatenation and phylogenetic inference (Table S3, also see Figure S2), totaling 770,922 nucleotide and 256,974 amino acid positions. After filtering out any gene where each taxon contained no more than 50% of the data as missing, a 229 gene data set resulted, totaling 334,317 nucleotide and 111,439 amino acid positions.

**Figure 1:**
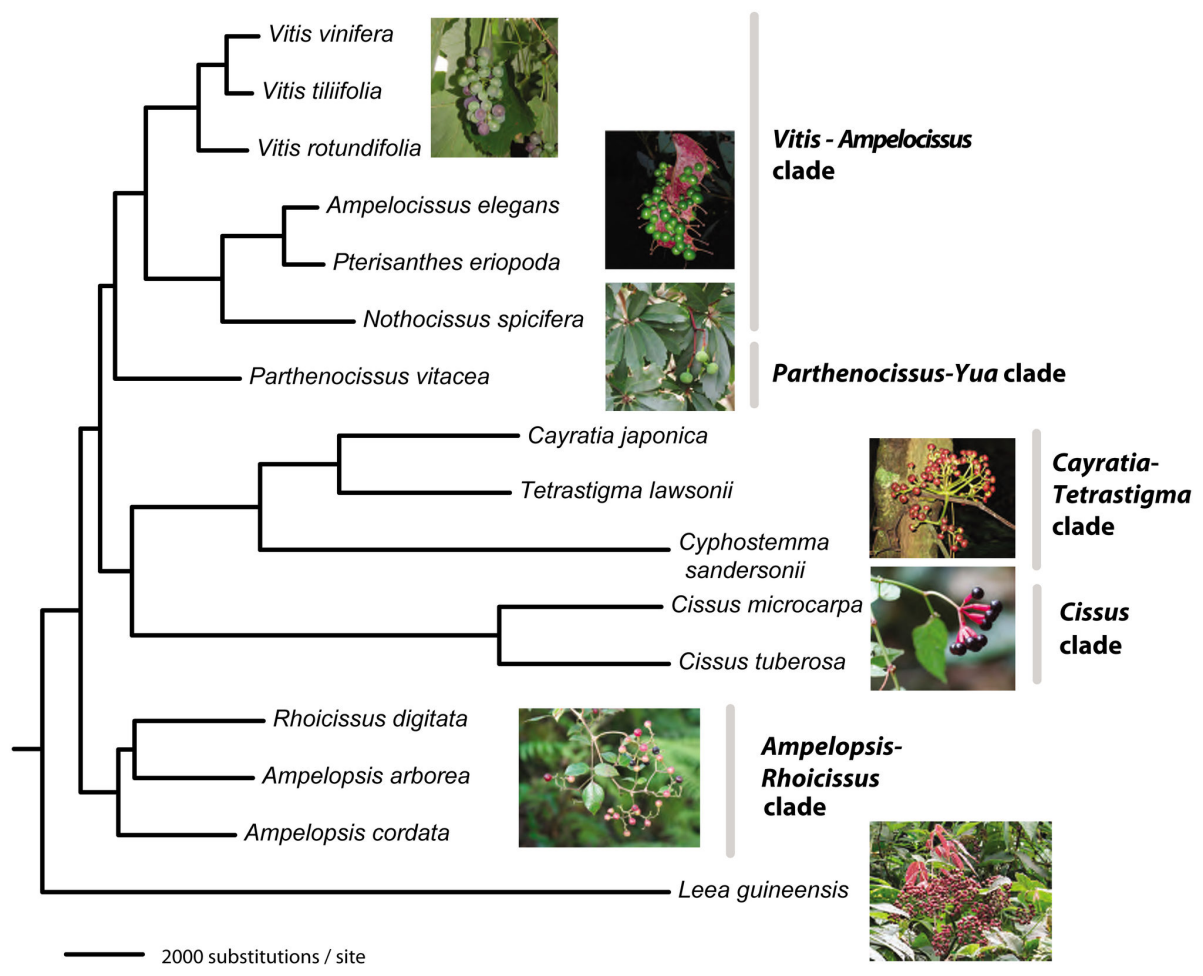
Maximum likelihood tree of Vitaceae using nucleotide sequences of 229 genes from the 15 transcriptomes of Vitaceae. The same topology was recovered from the 417 gene data set. Bootstrap support for all nodes was 100%, and posterior probabilities in the Bayesian inference for all nodes were 1.00.

**Figure 2:**
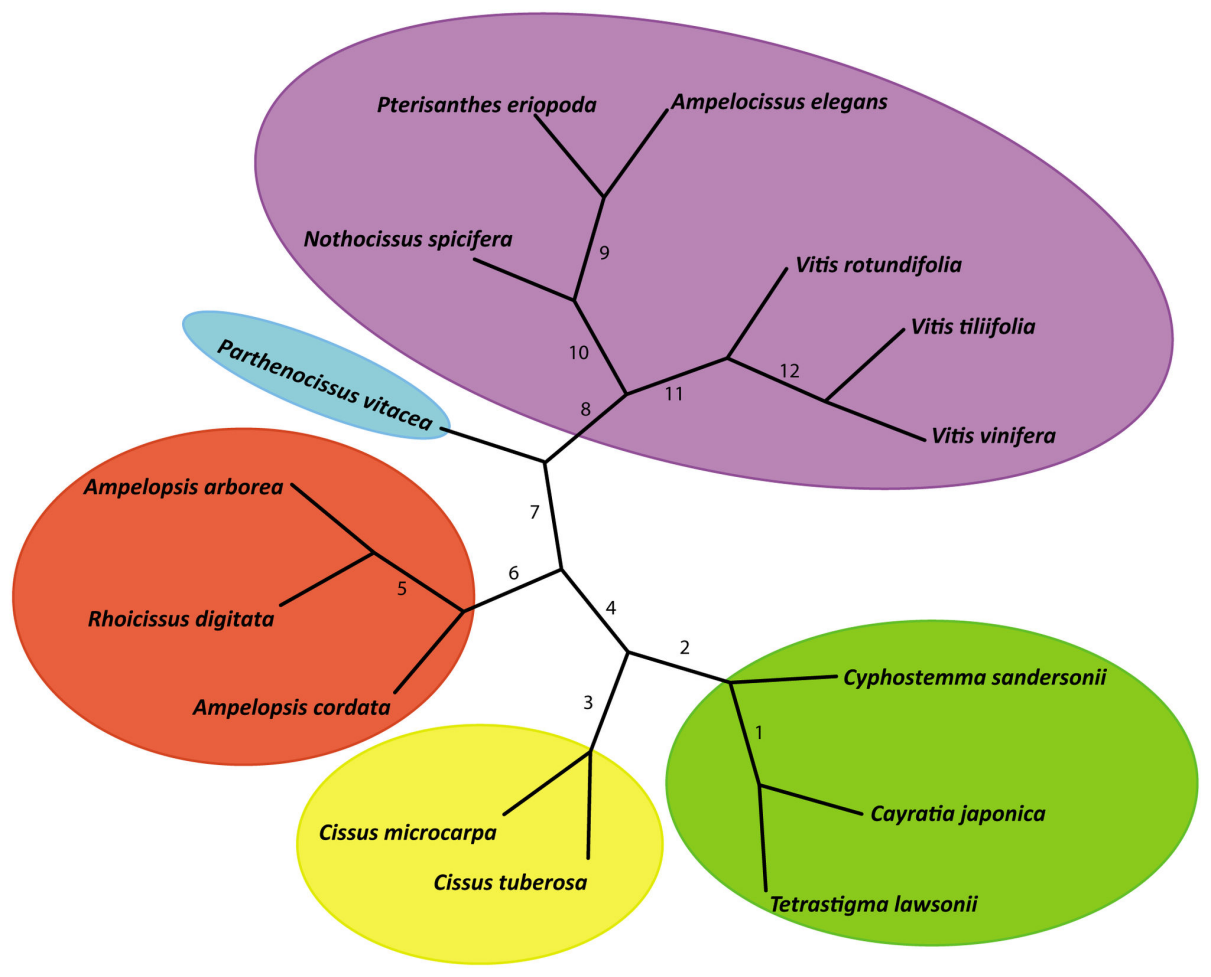
Unrooted tree of 15 species of Vitaceae based on nucleotide sequences of 229 genes. The node numbers correspond to those in Table S4.

Initial maximum likelihood analysis of the nucleotide sequences of the 417 gene matrix using PhyML [28] produced robust support for relationships of the grape family (Figure 1; all nodes with 100% bootstrap support values). However, to minimize the impact of missing data, we subsequently employed the 229 gene data set to explore various phylogenetic inference methods. Maximum likelihood estimates (ML [28,29]) and Bayesian inference (BI) with a phylogenetic mixture model [30] of the 229 gene data set also supported the topology shown in Figure 1. The maximum parsimony (MP) analyses [31], however, placed the *Cissus* clade at the base, even though the unrooted relationships within Vitaceae were identical with all three different analytical strategies (Figure 2). When we examined the data set closely, we noted that *Cissus* is the most divergent taxon within Vitaceae. The parsimony method has been known to be problematic under conditions of greatly unequal branch lengths, referred to as the long-branch attraction phenomenon [32]. Our analyses using maximum likelihood with both PhyML (28) and RAxML [29], and Bayesian inference [30] all yielded an identical topology of Vitaceae (Figure 1) that showed all nodes with 100% bootstrap support and posterior probabilities of 1.00, suggesting that all taxa of Vitaceae were represented by sufficient data to be reliably placed.

Thus, using the model-based analytical methods, we produced a transcriptome phylogeny (Figure 1) that supports the *Ampelopsis* – *Rhoicissus* clade as the basally diverged clade in Vitaceae. *Vitis*, *Ampelocissus* , *Pterisanthes* , and *Nothocissus* form a clade, which is sister to *Parthenocissus* . The taxa *Cissus*, *Cayratia* , *Cyphostemma* and *Tetrastigma* form a separate clade, with the latter three genera forming a subclade sister to core *Cissus*. These four genera possess two morphological synapomorphies: 4-merous flowers and very well-developed thick floral discs. This backbone relationship of Vitaceae is similar to the results of Ren et al. [3] using three chloroplast markers, but support values were relatively low for several major clades in that earlier study. It is of interest to mention that the deep clades, such as the *Parthenocissus* -*Vitis*-*Ampelocissus*-*Nothocissus*-*Pterisanthus* (PVANP) clade, as well as the clade of PVANP and *Cayratia* , *Tetrastigma* , *Cyphostemma* and *Cissus*, lack detectable morphological synapomorphies. Morphological convergence is the most likely reason for such a pattern at the deep level. All relationships at the shallower level are consistent with the results of the previous analyses of various clades of Vitaceae [4,6-8].

The biogeographic origin of the grape family has never been explored with analytical methods. With the phylogeny of Vitaceae unavailable at that time, in their seminal paper, Raven and Axelrod [33] considered Vitaceae as a relatively ancient family and proposed that it might have originated in the Laurasian region and subsequently reached the Southern Hemisphere subsequently. The first diverged clade, i.e., the *Ampelopsis* - *Rhoicissus* clade, consists of ca. 43 species disjunctly distributed over six continents (Asia, Europe, North America, South America, Africa, and Australia), and represents a rare example in angiosperms with such a widely disjunct distribution in both the Northern and the Southern Hemisphere. The *Ampelopsis* - *Rhoicissus* clade is composed of two distinct Laurasian lineages, each disjunct between the Old and the New World, and one Southern Hemisphere group with a Gondwana-like intercontinental disjunction: (Africa (Australia, and South America)). The biogeographic analyses of the 28 species sampled by Nie et al. [34] suggested that the *Ampelopsis* – *Rhoicissus* clade had an early diversification in the Northern Hemisphere and subsequently migrated into the Southern Hemisphere and diversified there. Our results also support the hypothesis that the primarily North Temperate grape genus *Vitis* forms a clade with the pantropical *Ampelocissus* , and the tropical Asian *Pterisanthes* and *Nothocissus* (Figure 1). This large clade of four genera consisting of the close relatives of grapes is sister to *Parthenocissus* , a North Temperate genus disjunct in eastern Asia and eastern North America. Even though a biogeographic analysis of the family is beyond the scope of the current paper, the establishment of the backbone phylogeny (Figure 1) will ultimately facilitate our inference of the family at the global scale and help elucidate the diversification processes involving both the temperate and tropical floristic elements. In particular, the placement of the *Ampelopsis* - *Rhoicissus* clade as the first diverged clade, the Northern Hemisphere taxa forming a grade, and the Southern Hemisphere taxa (e.g., *Rhoicissus* ) nested within the Northern Hemisphere grade (also see [34]) are consistent with the Northern Hemisphere origin of the family. A detailed biogeographic analysis with a broad taxon sampling scheme will be attempted in the near future.

**Figure 3:**
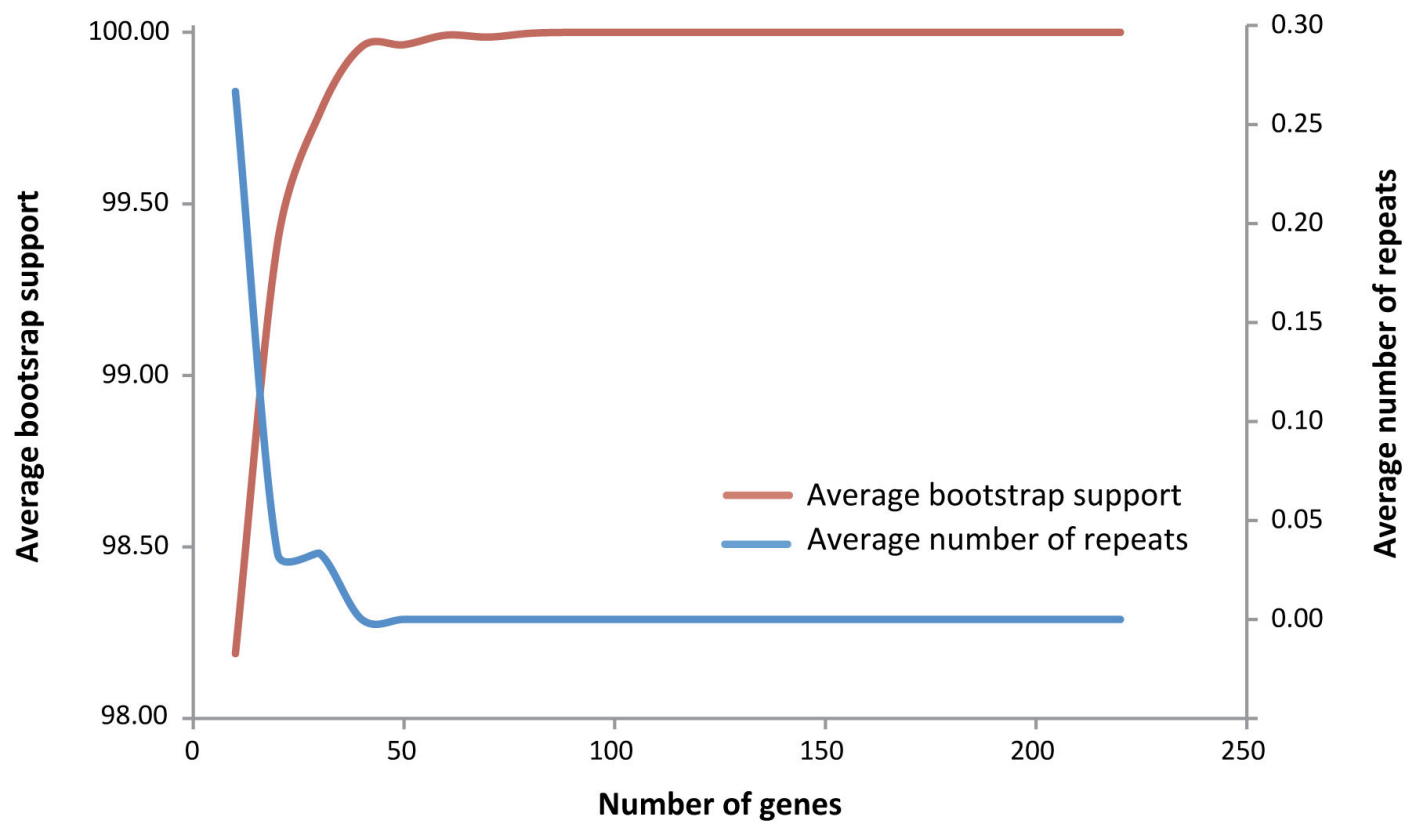
Average bootstrap support and the gene number in the phylogenetic analyses based on data from Table S4.

Given the strong support for the Vitaceae backbone phylogeny, we further tested its topological stability by producing new data sets via randomly reducing the gene number in multiples of 10, starting from the 229 gene tree. To automatically obtain bootstrap support scores on the nodes of large numbers of trees, we used the following strategy. For each specified gene number N, we obtained a random set of N genes from the 229 orthologous genes. Then we built an ML tree based on this set and compared the topology of the tree with that of the standard tree (Figure 2). If the topologies were the same, the set and tree were kept, or else they were discarded, and new sets and trees would be created and followed by comparison of the new topology to the 229 gene data set. The process was repeated until a tree with standard topology was obtained. For each N, the program built 30 trees based on 30 random sets of N genes. N was set to 10, 20, 30.. 220. The average numbers of repeats for each N were tabulated and plotted (Figure 3). With just 30 genes, all nodes had bootstrap support (BS) of more than 95% using the likelihood approach in PhyML; with 40 genes, all nodes had BS of at least 99% (Figure 2; Table S4). We also examined the average bootstrap support and the resampled nucleotide positions in the phylogenetic analyses to show the topological stability (Figure S3). Our results thus indicate that RNA-Seq [35], even with non-normalized transcriptomes, offers access to protein coding sequences very easily and quickly and represents a data-rich, accurate, and cost-effective source of orthologous sequences for phylogenetic inference.

Our data sets also showed that only 48 of the 229 gene trees had exactly the topology found in Figure 1, and in fact, the gene trees were quite diverse in topology. Nevertheless, the concatenated gene tree had all clades strongly supported. This result is reminiscent of the study of Rokas et al. [36], who demonstrated that concatenation of a sufficient number of randomly selected genes overwhelms conflicting signals present in different genes.

### Utility of transcriptome data for phylogenetic inference

**Figure 4:**
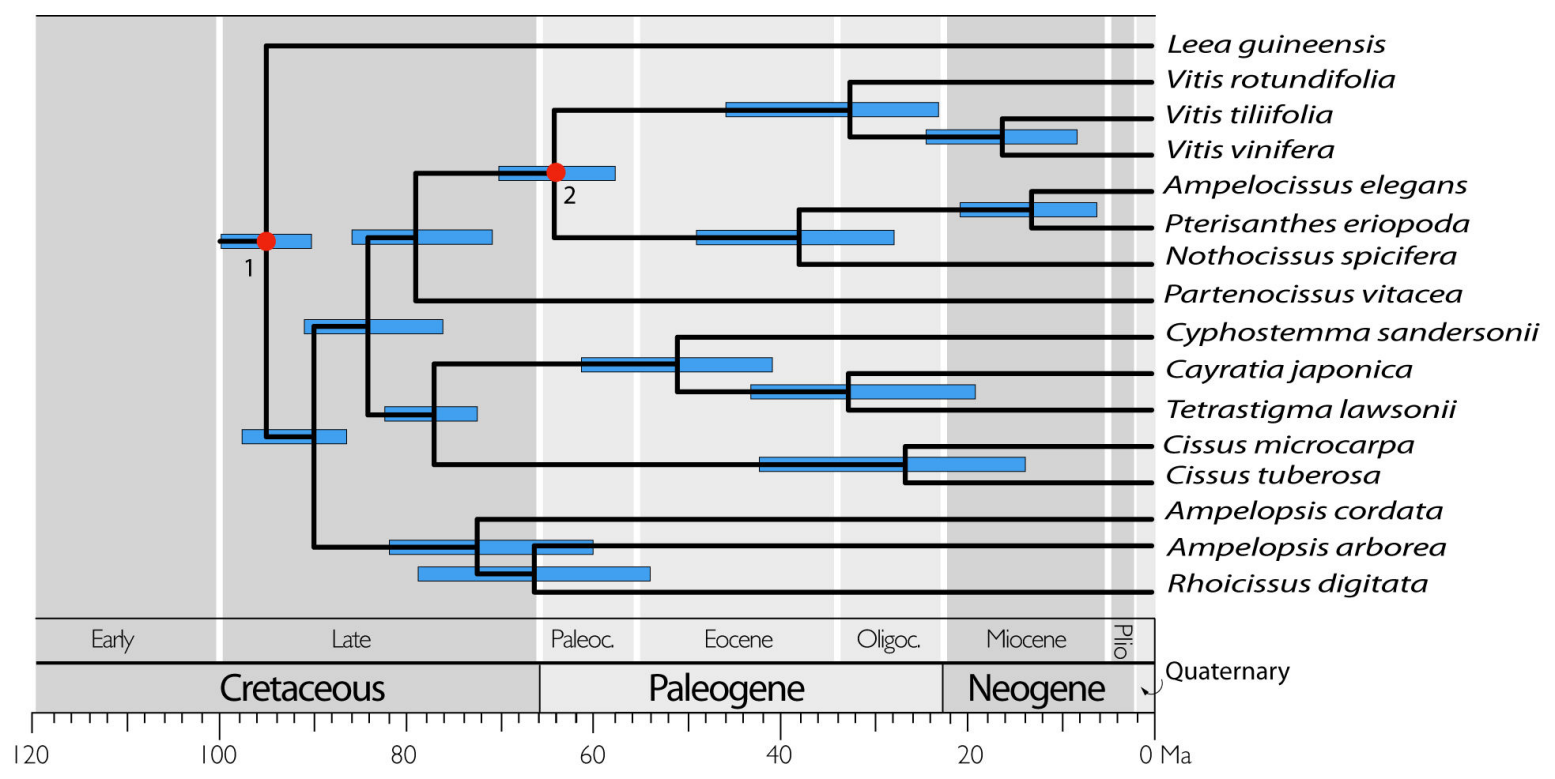
Divergence time estimation of Vitaceae clades using the program mcmctree in PAML and 4-fold degenerate sites. The red dots correspond to calibration points.

A practical disadvantage of using the transcriptome approach is that it requires high quality RNA from fresh material, while silica gel dried plant tissue samples and herbarium specimens will rarely yield good RNA. In fact, Hittinger et al. [20] have shown that large phylogenetic data matrices can be assembled accurately from even short (50 bp average) transcript sequences, so even non-optimal plant material, for example, that was preserved in “RNAlater” may eventually be used for transcriptome data generation. Our data demonstrate that the transcriptomes can yield resolution for previously difficult to resolve radiations, especially at the family level in plants, in the time frame since the mid-Cretaceous (Figure 4). This may be true, even though these are coding sequences, since their third positions and the 5’ and 3’ untranslated regions do evolve relatively rapidly [37]. Transcriptome data can effectively lead to identification of truly single copy transcripts and offer the conserved sequences necessary to generate primer pairs that can be used to amplify and sequence rapidly evolving intron regions for studies at and below the species level, generally without cloning steps [38]. The amplifications may then be standard ones followed by Sanger sequencing, or may be ones employed in the next-generation sequencing approaches generally referred to as targeted sequence capture [39,40]. Nevertheless, as the RNA-Seq approach is still relatively costly, extensive taxon sampling is not presently feasible. Our sampling in the grape family emphasized the backbone relationships and represents an example of what we can accomplish using transcriptomes and a first step toward resolving the deep phylogenetic relationships of Vitaceae.

Transcriptomic phylogenetic analyses do face some challenges due to the complications associated with pseudogenes and paralogous comparisons [38,41]. Because many plant lineages may have experienced reticulate evolution and allopolyploidy [42], it is also challenging to tease apart the plant diversification history given the hundreds of genes available. Our grape data set shows that transcriptome data can be an important source for phylogenetic inference. However, we may have been highly fortunate that the grape family was not seriously impacted by reticulate evolution in its early history (see Figure S4, based on the *Ks* value distribution of paralogs of species across the grape family), which allowed us to recover the highly robust topology (Figure 1).

With respect to data analyses, species tree approaches [43] may need to be explored more thoroughly and other partitioning strategies may be applied [44]. Testing and selecting genes with strong phylogenetic signals will be an important next step with our data set (see [45]). New analytical strategies will be needed to handle these large data sets, and to deal with realistic assumptions about the complications of molecular evolution as well as differences in nucleotide substitution rates. A number of common computer programs such as MrBayes [46], BEAST [47] and even PhyloBayes [44] cannot accommodate large data sets like ours with over 300,000 aligned nucleotide positions at present. Clearly, the systematic biology community needs to invest in the bioinformatics front more aggressively, as large data sets now are being generated at a rapid rate. Our study also demonstrates that the non-parametric parsimony method [31] may be misleading when handling genomic datasets with hundreds of thousands of characters when the sequence evolution is highly unequal across taxa in the study group. Furthermore, our case study on the grape family is within the time frame of 100 million years of evolution (Figure 4). If we move deeper into the time scale of the tree of life, we expect additional complications concerning homology of gene sequences. Nevertheless, plant biologists have experienced enormous difficulties in resolving deep relationships among taxa in the time frame of the last 100 million years, and our data demonstrate the power of transcriptome data over this evolutionary time scale.

## Materials and Methods

### Ethics Statement

No specific permits were required for the collection of samples as they were all grown in the greenhouse, which complied with all relevant regulations. None of the samples represents endangered or protected species.

### RNA extraction and transcriptome sequencing

Total RNA was isolated from finely ground mixed tissue samples of stems, leaves, tendrils and sometimes flowers of plants growing in the Botany Department greenhouse of the Smithsonian Institution. Voucher information for the species used is given in Table S1. We used the Sigma Spectrum™ Plant Total RNA Kit for the extractions. The transcriptome library construction and sequencing were performed at BGI and followed the protocols in Peng et al. [48].

### *De novo* assembly and transcript annotation

After we obtained raw sequencing data, we first filtered out reads of low quality, including cases of weak signal, large number of N’ s and PCR duplication. The reads with more than 40 bases of low quality, i.e., 71 or lower Illumina scores or with more than 20% of N (unknown) bases were all filtered out. Three software packages, Trinity [49], Velvet-Oases [50] and SOAPdenovo-Trans (http://soap.genomics.org.cn/SOAPdenovo-Trans.html) were evaluated for the initial assembly. The genes from the grape whole genome annotation were used as calibration to check the performance of the programs. We also used a gene set of conservative proteins in eukaryotes to evaluate the assemblies. After comparing the assemblies to check for completeness, redundancy, and the coverage of some essential or housekeeping genes in the grape genome, we selected the software SOAPdenovo-Trans as our assembler for its overall best performance.

After the *de novo* assembly of each sample with SOAPdenovo-Trans, we filtered out highly similar transcripts that may represent alternatively spliced transcripts. We then aligned the remaining transcripts to the reference grape genome in the Swiss-Prot database using BLAST [51] with the parameters “-e 1e-5 -F F -a 5”. The transcripts that could be aligned to reference sequences were selected and scanned to define coding regions (CDS). Length distribution of the coding regions extracted from the 14 transcriptomes and one reference genome (*Vitis vinifera*) of Vitaceae is shown in Figure S1.

### Gene orthologs

Self-to-self BLASTP [51] was conducted for all protein sequences with an E-value of 1E-5. We assigned a connection (edge) between two nodes (genes) if the aligned length was longer than 1/3 for both genes. An H-score that ranged from 0 to 100 was used to weight the edges. For genes G1 and G2, H-score is defined as Score (G1, G2) *100 / max(Score(G1, G1), Score(G2, G2)), where Score(A, B) is BLAST raw score of genes A and B.

To define gene families, we used average distance for a hierarchical clustering algorithm implemented in Hcluster_sg (part of TreeFam) [52]. It required the minimum edge weight (H-score) to be larger than 5 and the minimum edge density (total number of edges / theoretical number of edges) to be larger than 1/3. One-to-one single-copy orthologous gene families were then selected. The length distribution of 417 ortholog gene sequences (data including 6672 sequences, the total of 417 genes x 16 samples) is shown in Figure S2. The grape transcriptome sequence data have been deposited in GenBank (submission ID: Grape Transcriptome; submission content: Transcriptome analysis of 16 grapes; Submission: Grape Transcriptome; Created SUBMISSION: ACC = SRA081731 subid = 14992).

### Phylogenetic reconstruction

MUSCLE [53] was used to obtain multi-sequence alignments for each orthologous gene family. All alignments were concatenated for phylogenetic analyses using the optimality criteria of maximum parsimony (MP), maximum likelihood (ML), and Bayesian inference (BI), as implemented in PAUP 4.0b10 [31], PhyML 7.2.6 [28] and RAxML [29] and BayesPhylogenies [30], respectively. For the MP analyses, we used heuristic searches with tree-bisection-reconnection (TBR) branch swapping, MULTREES option on, and 1000 random additions. All characters were unordered and equally weighted, and gaps were treated as missing data in the analyses. For the ML analysis, the ML tree was calculated assuming a GTR + CAT model of sequence evolution. Robustness of inference was assessed by running 1000 fast bootstrap replicates. For the Bayesian analysis, we employed a joint model that accommodates both rate-heterotachy and pattern-heterogeneity as implemented in the program BayesPhylogenies [30]. We performed two runs of 2 million generations, sampling every 1000 generations, using 4 chains with the default heating scheme. After discarding the first 200,000 trees in the chain as a ‘‘burn-in’’ period, we sampled 1000 trees to ensure that successive trees in our sample were independent.

### Divergence time estimation

We used the program mcmctree in PAML [54] and 4-fold degenerate sites to estimate divergence time. The fossil record of Vitaceae is rich, and seed fossils can be differentiated at the generic level [55,56]. The oldest confirmed vitaceous seed fossil is unambiguously assigned to *Ampelocissus* s.l. ( *A* *. parvisemina* ) and dates back to the late Paleocene in North Dakota of North America [56]. Furthermore, *Ampelocissus* has been shown not to be monophyletic, but clearly forms a clade with *Vitis*, *Pterisanthes* , and *Nothocissus* [2,3]. The stem of the *Vitis*-*Ampelocissus*-*Pterisanthes*-*Nothocissus* clade was thus fixed at 58.5 ± 5.0 million yeas ago (Ma). For the root age of the family Vitaceae, Nie et al. [4,34] and Zecca et al. [57] fixed the split between Vitaceae and its sister lineage, *Leea*, as 85 ± 4.0 Ma based on the estimated age of 78-92 Ma by Wikström et al. [58]. However, Magallón and Castillo [59] reported a pre-Tertiary origin at 90.65 to 90.82 Ma for Vitaceae. The estimated ages from Magallón and Castillo [48] and Wikström et al. [58] are close, but the latter was criticized for using nonparametric rate smoothing and for calibrating the tree using only a single calibration point [50]. We herein use the estimate from Magallón and Castillo [59] and set the normal prior distribution of 90.7±1.0 Ma for the stem age of the family.

## Supporting Information

Figure S1 Length distribution of the coding regions extracted from the 14 transcriptomes and one reference genome (*Vitis vinifera*) of Vitaceae. Sample numbers are shown in Table S1.
(TIF)

Figure S2 Length distribution of 417 ortholog gene sequences (data include 6672 sequences, the total of 417 genes x 16 samples).
(TIF)

Figure S3 Average bootstrap support and the resampled nucleotide positions in the phylogenetic analyses to show the topological stability.
(TIF)

Figure S4 Ks value distributions for paralogs of Vitaceae species and the outgroup.
(TIF)

Table S1 Vitaceae species sampled for the grape transcriptome analyses. Voucher specimens are deposited at the US National Herbarium (US).
(DOCX)

Table S2 Statistical information of the transcriptomes of 15 species of Vitaceae and Leeaceae.
(DOCX)

Table S3 The 1:1:1 orthlog genes selected for phylogenetic analysis of the grape family.
(DOCX)

Table S4 Topological stability as estimated by bootstrap support of nodes with the maximum likelihood method by randomly reducing the gene number by 10, starting from the 229 gene data set.
(DOCX)

## References

[1] Wen J (2007) Vitaceae in Kubitzki K, The families and genera of vascular plants, vol. 9 Berlin: Springer-Verlag pp 466–478.

[2] Wen J, Nie Z-L, Soejima A, Meng Y (2007) Phylogeny of Vitaceae based on the nuclear *GAI1* gene sequences. Can J Bot 85: 731–745. doi:10.1139/B07-071.

[3] Ren H, Lu L-M, Soejima A, Luke Q, Zhang D-X et al. (2011) Phylogenetic analysis of the grape family (Vitaceae) based on the noncoding plastid *trnC-petN*, *trnH-psbA*, and *trnL-F* sequences. Taxon 60: 629–637.

[4] Nie ZL, Sun H, Chen ZD, Meng Y, Manchester SR et al. (2010) Molecular phylogeny and biogeographic diversification of *Parthenocissus* (Vitaceae) disjunct between Asia and North America. Am J Bot 97: 1342–1353. doi:10.3732/ajb.1000085. PubMed: 21616887. 21616887 10.3732/ajb.1000085

[5] Soejima A, Wen J (2006) Phylogenetic analysis of the grape family (Vitaceae) based on three chloroplast markers. Am J Bot 93: 278–287. doi:10.3732/ajb.93.2.278. PubMed: 21646189. 21646189 10.3732/ajb.93.2.278

[6] Lu L, Wen J, Chen Z (2012) A combined morphological and molecular phylogenetic analysis of *Parthenocissus* (Vitaceae) and taxonomic implications. Bot J Linn Soc, 168: 43–63. doi:10.1111/j.1095-8339.2011.01186.x.

[7] Lu L, Wang W, Chen Z, Wen J (2013) Phylogeny of the non-monophyletic *Cayratia* Juss. (Vitaceae) and implications for character evolution and biogeography. Mol Phylogenet Evol 68: 502–515. doi:10.1016/j.ympev.2013.04.023. PubMed: 23669013. 23669013 10.1016/j.ympev.2013.04.023

[8] Liu XQ, Ickert-Bond SM, Chen LQ, Wen J (2013) Molecular phylogeny of *Cissus* L. of Vitaceae (the grape family) and evolution of its pantropical intercontinental disjunctions. Mol Phylogenet Evol 66(1): 43–53. doi:10.1016/j.ympev.2012.09.003. PubMed: 23000818. 23000818 10.1016/j.ympev.2012.09.003

[9] Kocot KM, Cannon JT, Todt C, Citarella MR, Kohn AB et al. (2011) Phylogenomics reveals deep molluscan relationships. Nature 477: 452–456. doi:10.1038/nature10382. PubMed: 21892190. 21892190 10.1038/nature10382 PMC4024475

[10] Bell CD, Kutschker A, Arroyo MTK (2012) Phylogeny and diversification of Valerianaceae (Dipsacales) in the southern Andes. Mol Phylogenet Evol 63: 724–737. doi:10.1016/j.ympev.2012.02.015. PubMed: 22421085. 22421085 10.1016/j.ympev.2012.02.015

[11] Wen J, Plunkett GM, Mitchell A, Wagstaff S (2001) Evolution of Araliaceae: a phylogenetic analysis based on the ITS sequences of nrDNA. Syst Bot 26: 144–167.

[12] Plunkett GM, Wen J, Lowry PP (2004) Infrafamilial relationships in Araliaceae: Insights from nuclear (ITS) and plastid (*trnL*-*trnF*) sequence data. Plant Syst Evol 245: 1–39.

[13] Gernandt DS, Magallón S, Lopez GG, Flores OZ, Willyard A et al. (2008) Use of simultaneous analyses to guide fossil-based calibrations of Pinaceae phylogeny. Int J Plant Sci 169: 1086–1099. doi:10.1086/590472.

[14] Bremer B, Eriksson T (2009) Time tree of Rubiaceae: Phylogeny and dating the family, subfamilies and tribes. Int J Plant Sci 170: 766–793. doi:10.1086/599077.

[15] Franzke A, German D, Al-Shehbaz IA, Mummenhoff K (2009) *Arabidopsis* family ties: molecular phylogeny and age estimates in Brassicaceae. Taxon 58: 425–437.

[16] Olmstead RG, Zjhra ML, Lohmann LG, Grose SO, Eckert AJ (2009) A molecular phylogeny and classification of Bignoniaceae. Am J Bot 96: 1731–1743. doi:10.3732/ajb.0900004. PubMed: 21622359. 21622359 10.3732/ajb.0900004

[17] Nylander JAA, Olsson U, Alström P, Sanmartín I (2008) Accounting for phylogenetic uncertainty in biogeography: A Bayesian approach to dispersal-vicariance analysis of the thrushes (Aves: *Turdus*). Syst Biol 57: 257–268. doi:10.1080/10635150802044003. PubMed: 18425716. 18425716 10.1080/10635150802044003

[18] Ree RH, Smith SA (2008) Maximum likelihood inference of geographic range evolution by dispersal, local extinction, and cladogenesis. Syst Biol 57: 4–14. doi:10.1080/10635150701883881. PubMed: 18253896. 18253896 10.1080/10635150701883881

[19] Yu Y, Harris AJ, He X (2010) S-DIVA (Statistical Dispersal-Vicariance Analysis): A tool for inferring biogeographic histories. Mol Phylogenet Evol 56: 848–850. doi:10.1016/j.ympev.2010.04.011. PubMed: 20399277. 20399277 10.1016/j.ympev.2010.04.011

[20] Hittinger CT, Johnston M, Tossberg JT, Rokas A (2010) Leveraging skewed transcript abundance by RNA*-*Seq to increase the genomic depth of the tree of life. Proc Natl Acad Sci U S A 107: 1476–1481. doi:10.1073/pnas.0910449107. PubMed: 20080632. 20080632 10.1073/pnas.0910449107 PMC2824393

[21] Smith SA, Wilson NG, Goetz FE, Feehery C, Andrade SCS et al. (2011) Resolving the evolutionary relationships of molluscs with phylogenomic tools. Nature 480: 364–367. doi:10.1038/nature10526. PubMed: 22031330. 22031330 10.1038/nature10526

[22] Chiari Y, Cahais V, Galtier N, Delsuc F (2012) Phylogenomic analyses support the position of turtles as the sister group of birds and crocodiles (Archosauria). BMC Biol 10: 65. doi:10.1186/1741-7007-10-65. PubMed: 22839781. 22839781 10.1186/1741-7007-10-65 PMC3473239

[23] Barker MS, Kane NC, Matvienko M, Kozik A, Michelmore RW et al. (2008) Multiple paleopolyploidizations during the evolution of the Compositae reveal parallel patterns of duplicate gene retention after millions of years. Mol Biol Evol 25: 2445–2455. doi:10.1093/molbev/msn187. PubMed: 18728074. 18728074 10.1093/molbev/msn187 PMC2727391

[24] Barker MS, Vogel H, Schranz ME (2009) Paleopolyploidy in the Brassicales: Analyses of the Cleome transcriptome elucidate the history of genome duplications in Arabidopsis and other Brassicales. Genome Biol Evolution 1(1): 391–399. PubMed: 20333207. 10.1093/gbe/evp040 PMC2817432 20333207

[25] McKain MR, Wickett N, Zhang Y, Ayyampalayam S, McCombie WR et al. (2012) Phylogenomic analysis of transcriptome data elucidates co-occurrence of a paleopolyploid event and the origin of bimodal karyotypes in Agavoideae (Asparagaceae). Am J Bot 99: 397–406. doi:10.3732/ajb.1100537. PubMed: 22301890. 22301890 10.3732/ajb.1100537

[26] Cronn R, Knaus BJ, Liston A, Maughan PJ, Parks M et al. (2012) Targeted enrichment strategies for next-generation plant biology. Am J Bot 99: 291–311. doi:10.3732/ajb.1100356. PubMed: 22312117. 22312117 10.3732/ajb.1100356

[27] Jaillon O, Aury JM, Noel B, Policriti A, Clepet C et al. (2007) The grapevine genome sequence suggests ancestral hexaploidization in major angiosperm phyla. Nature 449: 463–467. doi:10.1038/nature06148. PubMed: 17721507. 17721507 10.1038/nature06148

[28] Guindon S, Dufayard J-F, Lefort V, Anisimova M, Hordijk W et al. (2010) New algorithms and methods to estimate maximum-likelihood phylogenies: assessing the performance of PhyML. Syst Biol 3.0 59: 307–321. 10.1093/sysbio/syq010 20525638

[29] Stamatakis A (2006) RAxML-VI-HPC: Maximum likelihood-based phylogenetic analyses with thousands of taxa and mixed models. Bioinformatics 22: 2688–2690. doi:10.1093/bioinformatics/btl446. PubMed: 16928733. 16928733 10.1093/bioinformatics/btl446

[30] Pagel M, Meade A (2004) A phylogenetic mixture model for detecting pattern-heterogeneity in gene sequence or character-state data. Syst Biol 53: 571–581. doi:10.1080/10635150490468675. PubMed: 15371247. 15371247 10.1080/10635150490468675

[31] Swofford DL (2003) PAUP*. Phylogenetic Analysis Using Parsimony (* and Other Methods), version 4. Sunderland, MA: Sinauer Associates.

[32] Felsenstein J (1978) Cases in which parsimony or compatibility methods will be positively misleading. Syst Zool 27: 401-410. doi:10.2307/2412923.

[33] Raven PH, Axelrod DI (1974) Angiosperm biogeography and past continental movements. Ann Mo Bot Gard 61: 539–673. doi:10.2307/2395021.

[34] Nie ZL, Sun H, Manchester SR, Meng Y, Luke Q et al. (2012) Evolution of the intercontinental disjunctions in six continents in the *Ampelopsis* clade of the grape family (Vitaceae). BMC Evol Biol 12: 17. doi:10.1186/1471-2148-12-17. 22316163 10.1186/1471-2148-12-17 PMC3299610

[35] Wang Z, Gerstein M, Snyder M (2009) RNA-Seq: A revolutionary tool for transcriptomics. Nat Rev Genet 10: 57–63. doi:10.1038/nrg2484. PubMed: 19015660. 19015660 10.1038/nrg2484 PMC2949280

[36] Rokas A, Williams BL, King N, Carroll SB (2003) Genome-scale approaches to resolving incongruence in molecular phylogenies. Nature 425: 798–804. doi:10.1038/nature02053. PubMed: 14574403. 14574403 10.1038/nature02053

[37] Whittall JB, Medina-Marino A, Zimmer EA, Hodges SA (2006) Generating single-copy nuclear gene data in a recent adaptive radiation. Mol Phylogenet Evol 39: 124–134. doi:10.1016/j.ympev.2005.10.010. PubMed: 16314114. 16314114 10.1016/j.ympev.2005.10.010

[38] Zimmer EA, Wen J (2012) Using nuclear gene data for plant phylogenetics: progress and prospects. Mol Phylogenet Evol 65: 774-785. doi:10.1016/j.ympev.2012.07.015. PubMed: 22842093. 22842093 10.1016/j.ympev.2012.07.015

[39] Faircloth BC, McCormack JE, Crawford NG, Harvey MG, Brumfield RT et al. (2012) Ultraconserved elements anchor thousands of genetic markers for target enrichment spanning multiple evolutionary timescales. Syst Biol 61: 717-726. doi:10.1093/sysbio/sys004. PubMed: 22232343. 22232343 10.1093/sysbio/sys004

[40] Grover CE, Salmon A, Wendel JF (2012) Targeted sequence capture as a powerful tool for evolutionary analysis. Am J Bot 99: 312–319. doi:10.3732/ajb.1100323. PubMed: 22268225. 22268225 10.3732/ajb.1100323

[41] Franssen SU, Shrestha RP, Bräutigam A, Bornberg-Bauer E, Weber APM (2011) Comprehensive transcriptome analysis of the highly complex *Pisum* *sativum* genome using next generation sequencing. BMC Genomics 12: 227. doi:10.1186/1471-2164-12-227. PubMed: 21569327. 21569327 10.1186/1471-2164-12-227 PMC3224338

[42] Soltis DE, Albert VA, Leebens-Mack J, Bell CD, Paterson AH et al. (2009) Polyploidy and angiosperm diversification. Am J Bot 96: 336–348. doi:10.3732/ajb.0800079. PubMed: 21628192. 21628192 10.3732/ajb.0800079

[43] Liu L, Yu L, Edwards SV (2010) A maximum pseudo-likelihood approach for estimating species trees under the coalescent model. BMC Evol Biol 10: 302. doi:10.1186/1471-2148-10-302. PubMed: 20937096. 20937096 10.1186/1471-2148-10-302 PMC2976751

[44] Lartillot N, Blanquart S, Lepage T (2012) PhyloBayes. p. 3.3, a Bayesian software for phylogenetic reconstruction and molecular dating using mixture models. Available: www.phylobayes.org. Accessed 2013 January 15. 10.1093/bioinformatics/btp368 19535536

[45] Salichos L, Rokas A (2013) Inferring ancient divergences requires genes with strong phylogenetic signals. Nature 497: 327-333. doi:10.1038/nature12130. PubMed: 23657258. 23657258 10.1038/nature12130

[46] Huelsenbeck JP, Ronquist R (2001) MRBAYES: Bayesian inference of phylogenetic trees. Bioinformatics 17: 754-755. doi:10.1093/bioinformatics/17.8.754. PubMed: 11524383. 11524383 10.1093/bioinformatics/17.8.754

[47] Drummond AJ, Rambaut A (2007) BEAST: Bayesian evolutionary analysis by sampling trees. BMC Evol Biol 7: 214. doi:10.1186/1471-2148-7-214. PubMed: 17996036. 17996036 10.1186/1471-2148-7-214 PMC2247476

[48] Peng Z, Cheng Y, Tan BC, Kang L, Tian Z et al. (2012) Comprehensive analysis of RNA-seq data reveals extensive RNA editing in a human transcriptome. Nat Biotechnol 30: 253–260. doi:10.1038/nbt.2122. PubMed: 22327324. 22327324 10.1038/nbt.2122

[49] Grabherr MG, Haas BJ, Yassour M, Levin JZ, Thompson DA et al. (2011) Full-length transcriptome assembly from RNA-seq data without a reference genome. Nat Biotechnol 29: 644–652. doi:10.1038/nbt.1883. PubMed: 21572440. 21572440 10.1038/nbt.1883 PMC3571712

[50] Schulz MH, Zerbino DR, Vingron M, Birney E (2012) Oases: Robust de novo RNA-seq assembly across the dynamic range of expression levels. Bioinformatics 28: 1086–1092. doi:10.1093/bioinformatics/bts094. PubMed: 22368243. 22368243 10.1093/bioinformatics/bts094 PMC3324515

[51] Altschul SF, Madden TL, Schäffer AA, Zhang J, Zhang Z et al. (1997) Gapped BLAST and PSI-BLAST: A new generation of protein database search programs. Nucleic Acids Res 25: 3389–3402. doi:10.1093/nar/25.17.3389. PubMed: 9254694. 9254694 10.1093/nar/25.17.3389 PMC146917

[52] Li H, Coghlan A, Ruan J, Coin LJ, Hériché JK et al. (2006) TreeFam: A curated database of phylogenetic trees of animal gene families. Nucleic Acids Res 34: D572–D580. doi:10.1093/nar/gkj118. PubMed: 16381935. 16381935 10.1093/nar/gkj118 PMC1347480

[53] Edgar RC (2004) MUSCLE: multiple sequence alignment with high accuracy and high throughput. Nucleic Acids Res 32: 1792–1797. doi:10.1093/nar/gkh340. PubMed: 15034147. 15034147 10.1093/nar/gkh340 PMC390337

[54] Yang Z (2007) PAML 4: Phylogenetic analysis by maximum likelihood. Mol Biol Evol 24: 1586–1591. doi:10.1093/molbev/msm088. PubMed: 17483113. 17483113 10.1093/molbev/msm088

[55] Chen I, Manchester SR (2007) Seed morphology of modern and fossil *Ampelocissus* (Vitaceae) and implications for phytogeography. Am J Bot 94: 1534–1553. doi:10.3732/ajb.94.9.1534. PubMed: 21636520. 21636520 10.3732/ajb.94.9.1534

[56] Chen I, Manchester SR (2011) Seed morphology of Vitaceae. Int J Plant Sci 172: 1–35. doi:10.1086/657283.

[57] Zecca G, Abbott JR, Sun WB, Spada A, Sala F et al. (2012) The timing and the mode of evolution of wild grapes (*Vitis*). Mol Phylogenet Evol 62: 736–747. doi:10.1016/j.ympev.2011.11.015. PubMed: 22138159. 22138159 10.1016/j.ympev.2011.11.015

[58] Wikström N, Savolainen V, Chase MW (2001) Evolution of the angiosperms: Calibrating the family tree. Proc R Soc Lond B 268: 2211–2220. doi:10.1098/rspb.2001.1782. PubMed: 11674868. 10.1098/rspb.2001.1782 PMC1088868 11674868

[59] Magallón SA, Castillo A (2009) Angiosperm diversification through time. Am J Bot 96: 349–365. doi:10.3732/ajb.0800060. PubMed: 21628193. 21628193 10.3732/ajb.0800060

