# Supplementary figures and images for: Transcriptome Sequences Resolve Deep Relationships of the Grape Family
Source: PLoS One. 2013 Sep 17;8(9):e74394. doi: 10.1371/journal.pone.0074394 (PMC3775763; doi:10.1371/journal.pone.0074394)

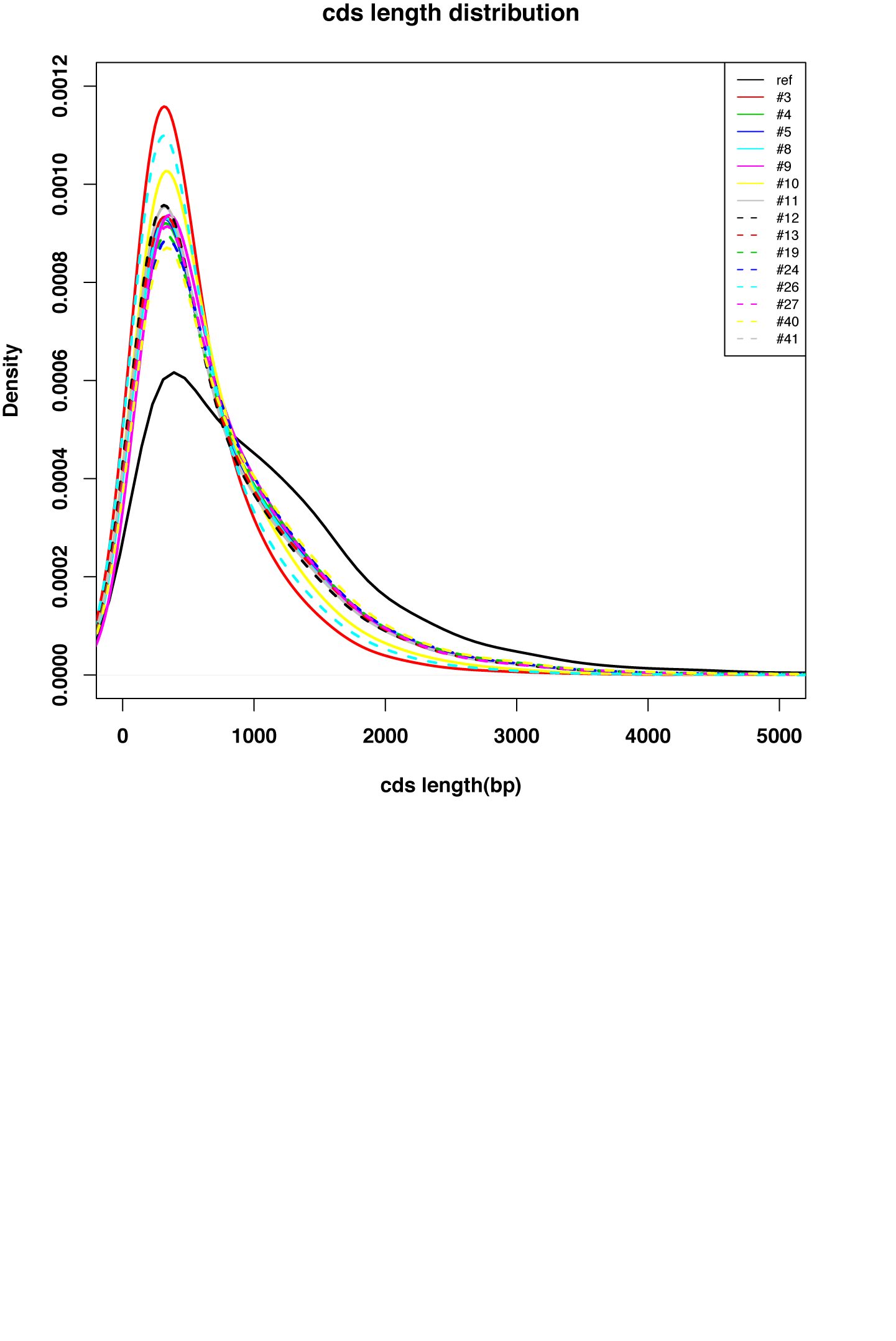

Supplement: Figure S1 — Length distribution of the coding regions extracted from the 14 transcriptomes and one reference genome (Vitis vinifera) of Vitaceae. Sample numbers are shown in Table S1. (TIF) [file pone.0074394.s001.tif]

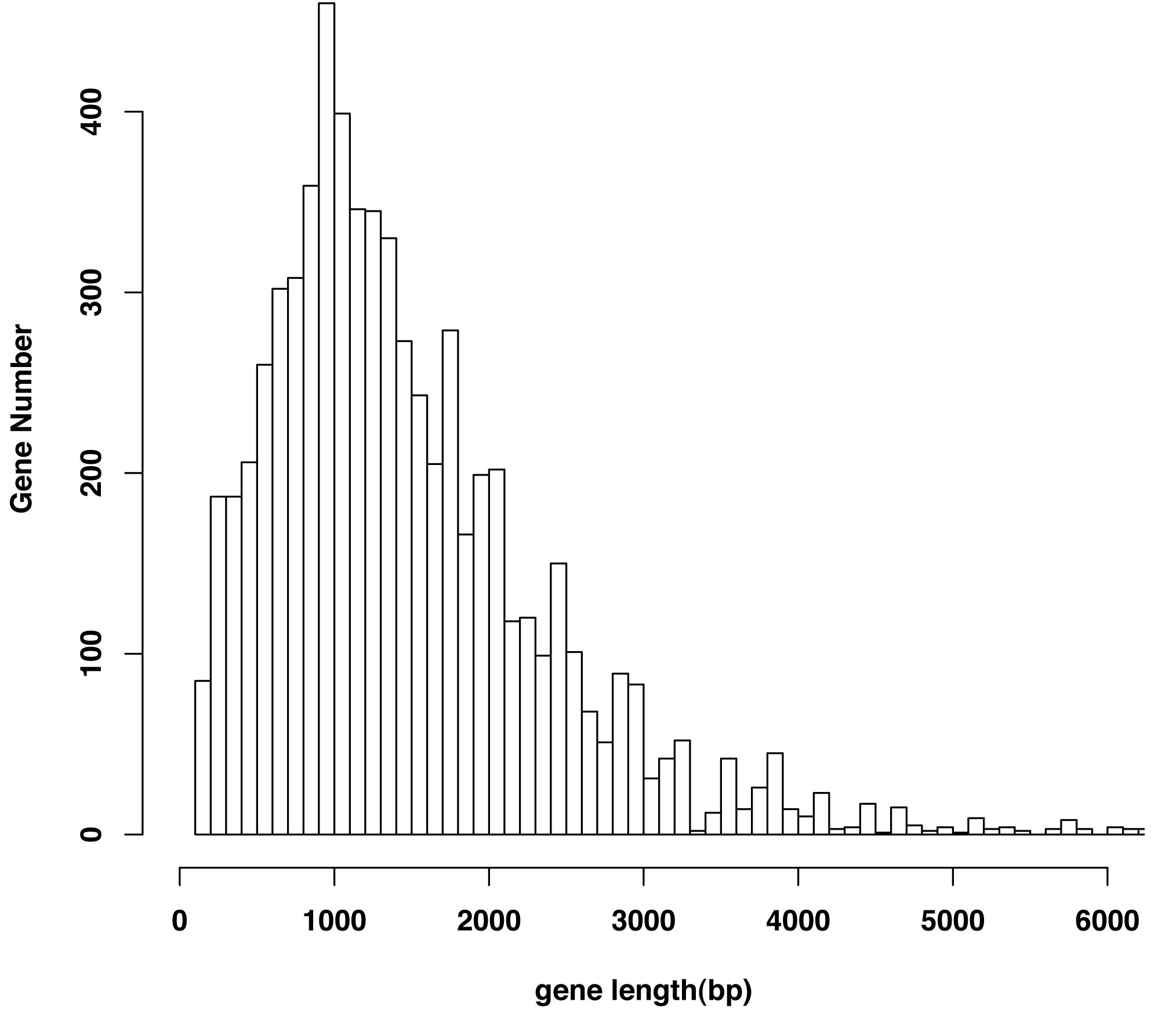

Supplement: Figure S2 — Length distribution of 417 ortholog gene sequences (data include 6672 sequences, the total of 417 genes x 16 samples). (TIF) [file pone.0074394.s002.tif]

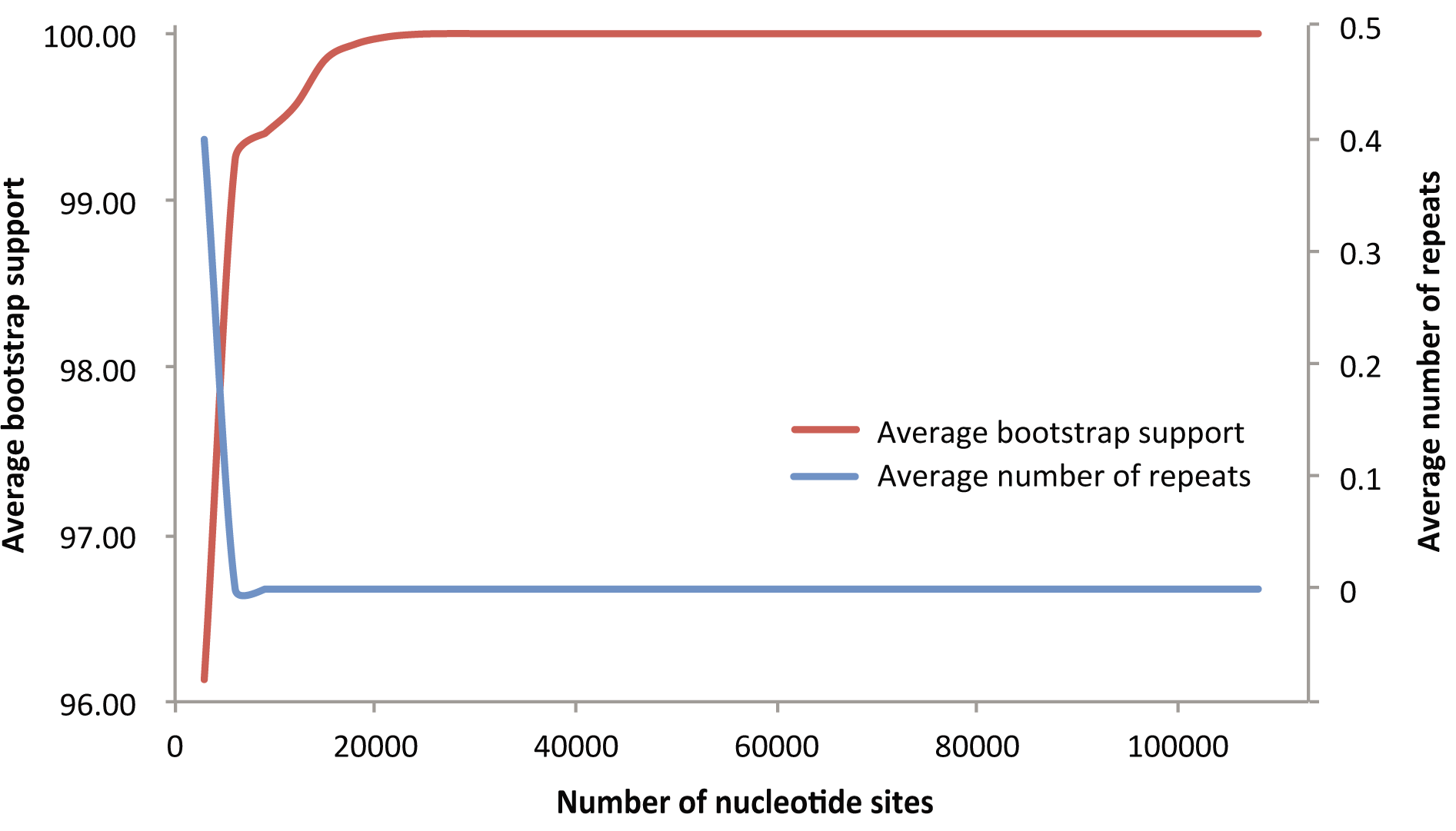

Supplement: Figure S3 — Average bootstrap support and the resampled nucleotide positions in the phylogenetic analyses to show the topological stability. (TIF) [file pone.0074394.s003.tif]

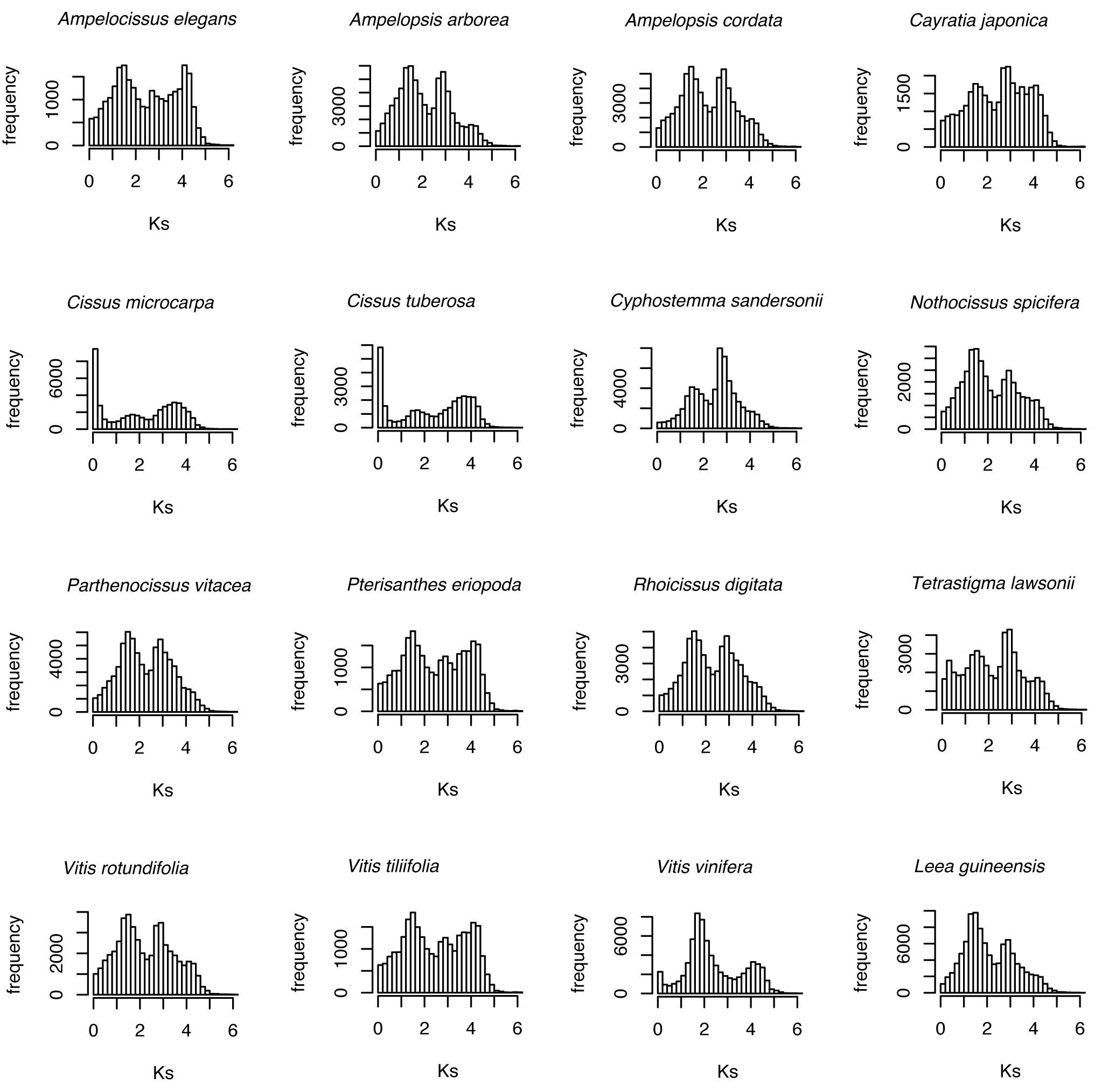

Supplement: Figure S4 — Ks value distributions for paralogs of Vitaceae species and the outgroup. (TIF) [file pone.0074394.s004.tif]
