# Supplementary material for: Transcriptome Sequences Resolve Deep Relationships of the Grape Family
Source: PLoS One. 2013 Sep 17;8(9):e74394. doi: 10.1371/journal.pone.0074394 (PMC3775763; doi:10.1371/journal.pone.0074394)
Supplement: Table S1 — Vitaceae species sampled for the grape transcriptome analyses. Voucher specimens are deposited at the US National Herbarium (US). (DOCX) [file pone.0074394.s005.docx]

**Table S1. Vitaceae species sampled for the grape transcriptome analyses. Voucher specimens are deposited at the US National Herbarium (US).**

| Species | RNA sample No. | Voucher No. |
| --- | --- | --- |
| *Ampelocissus elegans* | 41 | *Wen 11685* |
| *Ampelopsis arborea* | 08 | *Wen 12005* |
| *Ampelopsis cordata* | 13 | *Wen 12011* |
| *Cayratia japonica* | 10 | *Gerrath s.n.* |
| *Cissus microcarpa* | 03 | *Wen 11954* |
| *Cissus tuberosa* | 11 | *Gerrath s.n.* |
| *Cyphostemma sandersonii* | 40 | *Gerrath sn* |
| *Nothocissus spicifera* | 27 | *Wen 11675* |
| *Parthenocissus vitacea* | 04 | *Wen 11980* |
| *Pterisanthes eriopoda* | 26 | *Wen 11823* |
| *Rhoicissus digitata* | 05 | *Gerrath s.n.* |
| *Tetrastigma lawsonii* | 12 | *Wen 11680* |
| *Vitis rotundifolia* | 19 | *Wen 11963* |
| *Vitis tiliifolia* | 24 | *Wen 11948* |
| *Leea guineensis* | 09 | *Gerrath s.n.* |
