# Supplementary material for: Transcriptome Sequences Resolve Deep Relationships of the Grape Family
Source: PLoS One. 2013 Sep 17;8(9):e74394. doi: 10.1371/journal.pone.0074394 (PMC3775763; doi:10.1371/journal.pone.0074394)
Supplement: Table S2 — Statistical information of the transcriptomes of 15 species of Vitaceae and Leeaceae. (DOCX) [file pone.0074394.s006.docx]

**Table S2. Statistical information of the transcriptomes of 15 species of Vitaceae and Leeaceae.**

| Taxa | Raw | After filtering | Assembly size | Scaffold number | Filter | Longest scaffold (kb) | Scaffolds>1k | Scaffold N50 |
| --- | --- | --- | --- | --- | --- | --- | --- | --- |
|  | Total data (G) | Total data (G) |  |  |  |  |  |  |
| *Ampelocissus elegans* | 6.27 | 3.81 | 54,9362,61 | 73,862 | 50,322 | 19,122 | 18,103 | 1,566 |
| *Ampelopsis arborea* | 7.93 | 5.21 | 53,033,161 | 80,894 | 54,806 | 22,186 | 17,220 | 1,397 |
| *Ampelopsis cordata* | 8.01 | 4.36 | 51,590,113 | 78,695 | 52,476 | 21,030 | 16,852 | 1,411 |
| *Cayratia japonica* | 7.93 | 5.19 | 51,476,130 | 77,466 | 50,001 | 24,233 | 17,381 | 1,421 |
| *Cissus microcarpa* | 8.47 | 5.49 | 92,525,468 | 154,609 | 82,913 | 16,126 | 31,546 | 1,239 |
| *Cissus tuberosa* | 7.09 | 4.87 | 70,889,881 | 96,371 | 60,079 | 19,561 | 25,796 | 1,441 |
| *Cyphostemma sandersonii* | 6.19 | 4.23 | 50,496,327 | 75,129 | 46,296 | 23,906 | 16,660 | 1,414 |
| *Nothocissus spicifera* | 8.25 | 4 | 42,558,508 | 77,817 | 52,419 | 11,820 | 13,290 | 1,098 |
| *Parthenocissus vitacea* | 7.21 | 3.98 | 48,673,376 | 70,977 | 49,352 | 17,391 | 16,315 | 1,422 |
| *Pterisanthes eriopoda* | 8.46 | 4.78 | 52,674,049 | 75,003 | 50,715 | 24,992 | 17,839 | 1,512 |
| *Rhoicissus digitata* | 7.42 | 4.05 | 51,897,566 | 73,059 | 49,589 | 29,500 | 17,504 | 1,511 |
| *Tetrastigma lawsonii* | 8.32 | 5.53 | 54,372,601 | 86,553 | 56,423 | 24,180 | 17,490 | 1,374 |
| *Vitis rotundifolia* | 7.37 | 4.41 | 50,828,119 | 73,812 | 49,008 | 13,698 | 16,996 | 1,476 |
| *Vitis tiliifolia* | 8.26 | 4.96 | 52,435,505 | 75,629 | 50,552 | 14,673 | 17,704 | 1,492 |
| *Leea guineensis* | 8.35 | 4.71 | 58,915,254 | 88,109 | 59,068 | 18,558 | 18,900 | 1,466 |
