# Supplementary material for: Transcriptome Sequences Resolve Deep Relationships of the Grape Family
Source: PLoS One. 2013 Sep 17;8(9):e74394. doi: 10.1371/journal.pone.0074394 (PMC3775763; doi:10.1371/journal.pone.0074394)
Supplement: Table S3 — The 1:1:1 orthlog genes selected for phylogenetic analysis of the grape family. (DOCX) [file pone.0074394.s007.docx]

**Table S3. The 1:1:1 orthlog genes selected for phylogenetic analysis of the grape family.** *Protein ID refers to the *Arabidopsis thaliana* protein ID, which is the best fit of the *Vitis vinifera* genes from Jaillon et al. (2007). * represents the genes blasted to show similarities with *Arabidopsis thaliana* proteins located in the chloroplast, but they are chloroplast-like genes in the *Vitis* . ** represents the genes blasted to show similarities with *Arabidopsis thaliana* proteins located in mitochondrion, and are nuclear genes in *Vitis*. NA represents homolog genes not found from the *Arabidopsis thaliana* genome. Gene length is in nucleotides. The last column designates the genes with any taxon having missing data <50% (i.e, those taxa included in the 229 taxa data set derived from the 417 genes without any taxa with >50% missing data).

| Cluster ID | V.vinifera ID | Protein ID | Description | Gene length | missing_  ratio<50% |
| --- | --- | --- | --- | --- | --- |
| 297 | Vv10s0003g01550.t01 | AT4G18760.1 | receptor like protein 51 | 440 | yes |
| 1526 | Vv13s0084g00810.t01 | AT1G08315.1 | ARM repeat superfamily protein | 311 | no |
| 2286 | Vv11s0052g00530.t01 | AT5G05810.1 | RING/U-box superfamily protein | 457 | no |
| 2442 | Vv01s0011g01480.t01 | AT5G44800.1 | chromatin remodeling 4 | 2157 | no |
| 2519 | Vv07s0005g01810.t01 | AT1G11420.1 | DOMAIN OF UNKNOWN FUNCTION 724 2 | 1074 | yes |
| 2760 | Vv06s0004g07570.t01 | AT5G58410.1 | HEAT/U-box domain-containing protein | 1565 | no |
| 3003 | Vv02s0488g00040.t01 | AT5G47360.1 | Tetratricopeptide repeat (TPR)-like superfamily protein | 522 | no |
| 3041 | Vv00s0918g00010.t01 | AT2G17670.1 | Tetratricopeptide repeat (TPR)-like superfamily protein | 261 | yes |
| 3097 | Vv03s0063g01920.t01 | AT2G15980.1 | Tetratricopeptide repeat (TPR)-like superfamily protein | 364 | yes |
| 3114 | Vv16s0013g02120.t01 | AT4G30825.1 | Tetratricopeptide repeat (TPR)-like superfamily protein | 900 | no |
| 3119 | Vv07s0104g00910.t01 | AT2G30780.1 | Tetratricopeptide repeat (TPR)-like superfamily protein | 494 | yes |
| 4455 | Vv14s0006g02860.t01 | AT5G38380.1 | Protein of unknown function DUF6, transmembrane | 360 | no |
| 4640 | Vv03s0063g02480.t01 | AT4G34460.1 | GTP binding protein beta 1 | 377 | yes |
| 4643 | Vv14s0030g01350.t01 | AT3G05090.1 | Transducin/WD40 repeat-like superfamily protein | 802 | no |
| 4964 | Vv01s0010g00400.t01 | AT5G17370.1 | Transducin/WD40 repeat-like superfamily protein | 509 | no |
| 4983 | Vv17s0000g08930.t01 | AT1G73720.1 | transducin family protein / WD-40 repeat family protein | 514 | yes |
| 5030 | Vv13s0084g00110.t01 | AT3G10670.1 | non-intrinsic ABC protein 7 | 321 | yes |
| 5491 | Vv05s0062g00860.t01 | AT2G32000.1 | DNA topoisomerase, type IA, core | 821 | no |
| 7199 | Vv11s0037g01040.t01 | AT2G23890.1 | HAD-superfamily hydrolase, subfamily IG, 5'-nucleotidase | 153 | no |
| 7214 | Vv14s0006g01840.t01 | AT5G37930.1 | Protein with RING/U-box and TRAF-like domains | 633 | no |
| 7314 | Vv15s0021g01530.t01 | AT1G02120.1 | GRAM domain family protein | 639 | yes |
| 7358 | Vv05s0077g01830.t01 | AT3G16785.1 | phospholipase D P1 | 1112 | no |
| 7500 | Vv06s0061g01590.t01 | AT3G44600.1 | cyclophilin71 | 594 | no |
| 7507 | Vv19s0014g03350.t01 | AT3G15520.1 | Cyclophilin-like peptidyl-prolyl cis-trans isomerase family protein | 331 | yes |
| 7514 | Vv19s0027g01660.t01 | AT1G53720.1 | cyclophilin 59 | 638 | yes |
| 7557 | Vv18s0075g00210.t01 | AT1G35620.1 | PDI-like 5-2 | 396 | yes |
| 7725 | Vv14s0081g00150.t01 | AT3G04710.1 | ankyrin repeat family protein | 458 | yes |
| 7857 | Vv12s0028g03360.t01 | AT2G20635.1 | ATP binding;protein kinases;protein serine/threonine kinases | 552 | no |
| 7881 | Vv06s0009g01060.t01 | AT5G20930.1 | Protein kinase superfamily protein | 679 | yes |
| 8268 | Vv03s0038g00860.t01 | AT4G38900.1 | Basic-leucine zipper (bZIP) transcription factor family protein | 676 | no |
| 8293 | Vv07s0191g00210.t01 | AT4G01020.1 | helicase domain-containing protein / IBR domain-containing protein / zinc finger protein-related | 1737 | no |
| 8357 | Vv05s0029g00210.t01 | AT1G04860.1 | ubiquitin-specific protease 2 | 66 | yes |
| 8439 | Vv04s0044g02020.t01 | AT5G65930.2 | kinesin-like calmodulin-binding protein (ZWICHEL) | 1268 | no |
| 8631 | Vv10s0116g00830.t01 | AT1G61850.1 | phospholipases;galactolipases | 1286 | no |
| 9461 | Vv15s0021g02260.t01 | AT4G00030.1 | Plastid-lipid associated protein PAP / fibrillin family protein | 255 | yes |
| 9520 | Vv05s0020g01470.t01 | AT3G16565.2 | alanine-tRNA ligases;nucleic acid binding;ligases, forming aminoacyl-tRNA and related compounds;nucleotide binding;ATP binding | 256 | yes |
| 9896 | Vv15s0046g02870.t01 | AT3G61320.1 | Bestrophin-like protein | 419 | no |
| 10492 | Vv15s0046g02250.t01 | AT1G01510.1 | NAD(P)-binding Rossmann-fold superfamily protein | 644 | no |
| 10506 | Vv15s0021g01120.t01 | AT1G02160.2 | Cox19 family protein (CHCH motif) | 70 | yes |
| 10592 | Vv11s0078g00440.t01 | AT5G16780.1 | SART-1 family | 944 | yes |
| 11289 | Vv18s0001g09110.t01 | AT5G43190.1 | Galactose oxidase/kelch repeat superfamily protein | 521 | no |
| 11337 | Vv01s0150g00110.t01 | AT5G61910.3 | DCD (Development and Cell Death) domain protein | 489 | no |
| 11351 | Vv06s0009g02110.t01 | AT5G26960.1 | Galactose oxidase/kelch repeat superfamily protein | 333 | yes |
| 11703 | Vv13s0019g05240.t01 | AT3G10500.1 | NAC domain containing protein 53 | 559 | yes |
| 11945 | Vv16s0022g01340.t01 | AT4G25290.1 | DNA photolyases;DNA photolyases | 688 | no |
| *12052 | Vv06s0009g02300.t01 | AT1G08490.1 | chloroplastic NIFS-like cysteine desulfurase | 463 | no |
| 12277 | Vv09s0002g07070.t01 | AT1G80070.1 | Pre-mRNA-processing-splicing factor | 2347 | no |
| 12530 | Vv04s0008g01780.t01 | AT5G47430.1 | DWNN domain, a CCHC-type zinc finger | 795 | no |
| 12531 | Vv00s0341g00020.t01 | AT5G47430.1 | DWNN domain, a CCHC-type zinc finger | 828 | no |
| 12532 | Vv16s0098g01750.t01 | AT5G53020.1 | Ribonuclease P protein subunit P38-related | 818 | yes |
| 12646 | Vv05s0020g04590.t01 | AT4G14570.1 | acylaminoacyl-peptidase-related | 822 | no |
| 12709 | Vv04s0023g01840.t01 | AT3G50870.1 | GATA type zinc finger transcription factor family protein | 240 | no |
| 12816 | Vv16s0039g01430.t01 | AT5G16690.1 | origin recognition complex subunit 3 | 371 | no |
| 13013 | Vv15s0048g01810.t01 | AT3G61080.1 | Protein kinase superfamily protein | 330 | yes |
| 13050 | Vv01s0011g04500.t01 | AT1G15140.1 | FAD/NAD(P)-binding oxidoreductase | 263 | no |
| 13448 | Vv17s0000g04170.t01 | AT3G18524.1 | MUTS homolog 2 | 945 | no |
| 13653 | Vv02s0025g02400.t01 | AT4G10760.1 | mRNAadenosine methylase | 764 | yes |
| *13679 | Vv06s0004g07080.t01 | AT5G58510.1 | unknown protein | 954 | no |
| 13686 | Vv12s0028g02620.t01 | AT2G42390.1 | protein kinase C substrate, heavy chain-related | 205 | no |
| 13715 | Vv00s0253g00060.t01 | AT5G60200.1 | TARGET OF MONOPTEROS 6 | 352 | no |
| 13848 | Vv15s0048g00470.t01 | AT3G60740.1 | ARM repeat superfamily protein | 1269 | no |
| 13861 | Vv04s0008g00910.t01 | AT5G26040.2 | histone deacetylase 2 | 359 | no |
| 13883 | Vv03s0088g00280.t01 | AT1G50000.1 | methyltransferases | 454 | no |
| 14019 | Vv07s0005g04490.t01 | AT2G20330.1 | Transducin/WD40 repeat-like superfamily protein | 746 | yes |
| 14131 | Vv06s0009g02520.t01 | AT5G22010.1 | replication factor C1 | 864 | no |
| 14156 | Vv14s0083g00750.t01 | AT5G15680.1 | ARM repeat superfamily protein | 2149 | no |
| 14376 | Vv19s0090g01820.t01 | AT1G55930.1 | CBS domain-containing protein / transporter associated domain-containing protein | 567 | no |
| 14436 | Vv13s0067g02190.t01 | AT3G10980.1 | PLAC8 family protein | 711 | no |
| 14492 | Vv01s0011g00530.t01 | AT2G03590.1 | ureide permease 1 | 397 | no |
| *14555 | Vv03s0091g01100.t01 | AT1G49975.1 | INVOLVED IN: photosynthesis; LOCATED IN: photosystem I, chloroplast, thylakoid membrane; | 119 | yes |
| 14556 | Vv10s0003g04600.t01 | AT1G30460.1 | cleavage and polyadenylation specificity factor 30 | 673 | yes |
| 14725 | Vv05s0020g02070.t01 | AT3G21480.1 | BRCT domain-containing DNA repair protein | 1224 | no |
| 14854 | Vv14s0171g00520.t01 | AT5G40740.1 | unknown protein; | 747 | no |
| 14916 | Vv07s0031g01770.t01 | AT2G21860.1 | violaxanthin de-epoxidase-related | 532 | no |
| 14967 | Vv08s0007g05330.t01 | AT3G56990.1 | embryo sac development arrest 7 | 701 | no |
| 14973 | Vv08s0007g00520.t01 | AT3G09310.1 | Protein of unknown function DUF37 (InterPro:IPR002696); | 143 | yes |
| 15071 | Vv08s0056g00040.t01 | AT5G24710.1 | Transducin/WD40 repeat-like superfamily protein | 1824 | no |
| 15171 | Vv07s0005g02430.t01 | AT4G01660.1 | ABC transporter 1 | 583 | no |
| 15228 | Vv08s0056g01180.t01 | AT3G09180.1 | Mediator complex subunit Med27 (InterPro:IPR021627) | 419 | yes |
| 15255 | Vv07s0005g01460.t01 | AT2G47390.1 | Prolyl oligopeptidase family protein | 913 | yes |
| 15486 | Vv05s0077g00480.t01 | AT4G13550.1 | triglyceride lipases;triglyceride lipases | 654 | no |
| **15794 | Vv14s0036g00480.t01 | AT3G02660.1 | Tyrosyl-tRNA synthetase, class Ib, bacterial/mitochondrial | 485 | yes |
| 15841 | Vv17s0000g04440.t01 | AT5G23080.1 | SWAP (Suppressor-of-White-APricot)/surp domain-containing protein | 997 | no |
| 15861 | Vv02s0025g03970.t01 | AT1G12800.1 | Nucleic acid-binding, OB-fold-like protein | 773 | no |
| 16006 | Vv19s0015g01810.t01 | AT5G56240.1 | hapless 8 | 1391 | no |
| *16078 | Vv08s0040g00480.t01 | AT3G56810.1 | unknown protein;LOCATED IN: chloroplast; | 339 | yes |
| 16097 | Vv08s0007g00050.t01 | AT5G02520.1 | SANT associated (InterPro:IPR015216); | 463 | no |
| 16111 | Vv11s0037g00300.t01 | AT4G30600.1 | signal recognition particle receptor alpha subunit family protein | 616 | yes |
| 16333 | Vv07s0129g01060.t01 | AT5G67290.1 | FAD-dependent oxidoreductase family protein | 420 | yes |
| 16345 | Vv11s0103g00650.t01 | AT4G29940.1 | pathogenesis related homeodomain protein A | 727 | no |
| 16374 | Vv01s0011g01060.t01 | AT4G19610.1 | nucleotide binding;nucleic acid binding;RNA binding | 808 | no |
| 16422 | Vv11s0016g03230.t01 | AT2G19950.1 | golgin candidate 1 | 712 | yes |
| 16566 | Vv02s0012g01210.t01 | AT4G17830.1 | Peptidase M20/M25/M40 family protein | 433 | no |
| 16577 | Vv07s0031g00840.t01 | AT5G66810.1 | CONTAINS InterPro DOMAIN/s: CTLH, C-terminal LisH motif (InterPro:IPR006595); | 710 | no |
| 16621 | Vv04s0008g02700.t01 | AT5G24840.1 | tRNA (guanine-N-7) methyltransferase | 252 | yes |
| 16654 | Vv04s0023g00150.t01 | AT3G44370.1 | Membrane insertion protein, OxaA/YidC with tetratricopeptide repeat domain | 572 | yes |
| 16670 | Vv07s0005g01440.t01 | AT4G02030.1 | Vps51/Vps67 family (components of vesicular transport) protein | 782 | yes |
| 16769 | Vv01s0010g01440.t01 | AT3G19840.1 | pre-mRNA-processing protein 40C | 829 | yes |
| 16956 | Vv07s0005g04370.t01 | AT2G20390.1 | unknown protein; | 191 | yes |
| 17181 | Vv05s0020g01250.t01 | AT4G15840.1 | BTB/POZ domain-containing protein | 825 | yes |
| 17307 | Vv08s0007g06190.t01 | AT3G57890.1 | Tubulin binding cofactor C domain-containing protein | 568 | yes |
| 17485 | Vv06s0004g03370.t01 | NA | Phosphatidylinositol transfer protein(IPR001666); | 292 | yes |
| 17561 | Vv11s0052g00080.t01 | AT2G19270.1 | Mitotic checkpoint protein PRCC, C-terminal (InterPro:IPR018800); | 367 | yes |
| 17593 | Vv13s0084g00170.t01 | AT5G45140.1 | nuclear RNA polymerase C2 | 753 | no |
| 17607 | Vv12s0035g00640.t01 | AT4G06599.1 | ubiquitin family protein | 327 | yes |
| 17609 | Vv17s0000g05200.t01 | AT4G26190.1 | Haloacid dehalogenase-like hydrolase (HAD) superfamily protein | 652 | no |
| 17637 | Vv03s0038g01240.t01 | AT3G24200.2 | FAD/NAD(P)-binding oxidoreductase family protein | 536 | yes |
| 17760 | Vv06s0009g01760.t01 | AT5G21040.1 | F-box protein 2 | 592 | yes |
| **17789 | Vv03s0091g00220.t01 | AT3G19508.1 | unknown protein; LOCATED IN: mitochondrion; | 82 | yes |
| 17824 | Vv02s0012g01750.t01 | AT1G32340.1 | NDR1/HIN1-like 8 | 662 | no |
| 17850 | Vv14s0128g00850.t01 | AT1G55040.1 | zinc finger (Ran-binding) family protein | 821 | no |
| 17853 | Vv19s0014g00970.t01 | AT1G53460.1 | Ran BP2/NZF zinc finger-like superfamily protein | 330 | no |
| 17996 | Vv00s0227g00060.t01 | AT2G40360.1 | Transducin/WD40 repeat-like superfamily protein | 695 | yes |
| 18027 | Vv12s0028g03940.t01 | AT1G32750.1 | HAC13 protein (HAC13) | 1913 | yes |
| 18060 | Vv18s0001g01830.t01 | AT1G21640.1 | NAD kinase 2 | 1027 | no |
| 18226 | Vv02s0025g00230.t01 | NA | NA | 87 | yes |
| 18301 | Vv02s0025g03280.t01 | AT1G63770.3 | Peptidase M1 family protein | 495 | yes |
| 18334 | Vv12s0057g00230.t01 | AT1G12470.1 | zinc ion binding | 986 | yes |
| 18496 | Vv09s0002g08940.t01 | AT1G80680.1 | SUPPRESSOR OF AUXIN RESISTANCE 3 | 790 | no |
| 18543 | Vv10s0003g04680.t01 | AT3G29130.1 | Domain of unknown function KxDL (InterPro:IPR019371); | 123 | yes |
| 18555 | Vv10s0116g01560.t01 | AT4G28200.1 | unknown protein;INVOLVED IN: RNA processing; | 652 | yes |
| 18623 | Vv14s0108g01370.t01 | AT5G39410.1 | Saccharopine dehydrogenase | 451 | no |
| 18713 | Vv19s0014g02260.t01 | AT5G53930.1 | unknown protein; LOCATED IN: chloroplast; | 597 | yes |
| 18774 | Vv09s0002g02290.t01 | AT1G72990.1 | beta-galactosidase 17 | 708 | yes |
| 18791 | Vv06s0080g00510.t01 | AT2G27460.1 | sec23/sec24 transport family protein | 744 | yes |
| 18905 | Vv13s0064g01080.t01 | AT1G77090.1 | Mog1/PsbP/DUF1795-like photosystem II reaction center PsbP family protein | 247 | yes |
| 18962 | Vv00s0742g00030.t01 | AT5G65000.1 | Nucleotide-sugar transporter family protein | 327 | yes |
| 18981 | Vv02s0012g00030.t01 | AT5G47090.1 | Protein of unknown function DUF2052, coiled-coil (InterPro:IPR018613); | 343 | no |
| 19021 | Vv05s0077g01770.t01 | AT3G16810.1 | pumilio 24 | 662 | yes |
| 19047 | Vv14s0030g00020.t01 | NA | NA | 161 | yes |
| 19105 | Vv06s0009g03530.t01 | AT4G03250.1 | Homeodomain-like superfamily protein | 528 | no |
| 19129 | Vv09s0002g02510.t01 | AT3G21290.1 | dentin sialophosphoprotein-related | 1253 | no |
| 19193 | Vv04s0043g00740.t01 | AT5G18410.1 | transcription activators | 1261 | no |
| 19236 | Vv03s0038g04610.t01 | AT4G25730.1 | FtsJ-like methyltransferase family protein | 851 | no |
| 19266 | Vv08s0217g00040.t01 | AT3G08960.1 | ARM repeat superfamily protein | 564 | no |
| 19297 | Vv14s0036g01060.t01 | AT5G28960.1 | unknown protein; LOCATED IN: endomembrane system; | 565 | yes |
| 19376 | Vv13s0067g02940.t01 | AT3G55850.2 | Amidohydrolase family | 578 | no |
| 19390 | Vv05s0029g00550.t01 | AT4G30990.2 | ARM repeat superfamily protein | 1587 | no |
| 19504 | Vv14s0030g02240.t01 | AT5G16940.1 | carbon-sulfur lyases | 201 | no |
| 19597 | Vv03s0088g00450.t01 | AT1G50030.1 | target of rapamycin | 2937 | no |
| 19654 | Vv13s0067g03320.t01 | AT3G55000.1 | tonneau family protein | 265 | yes |
| 19668 | Vv04s0008g03620.t01 | AT5G11560.1 | catalytics | 987 | yes |
| 19731 | Vv08s0007g00460.t01 | AT5G02250.1 | Ribonuclease II/R family protein | 720 | yes |
| 19748 | Vv12s0028g00640.t01 | AT1G06560.1 | NOL1/NOP2/sun family protein | 586 | no |
| 19752 | Vv06s0009g01580.t01 | AT5G21070.1 | unknown protein; | 228 | yes |
| 19763 | Vv01s0011g05710.t01 | AT2G01690.2 | ARM repeat superfamily protein | 727 | no |
| *19783 | Vv13s0067g01340.t01 | AT3G56010.1 | unknown protein;LOCATED IN: chloroplast thylakoid membrane; | 253 | yes |
| 19835 | Vv18s0001g06830.t01 | AT1G22800.1 | S-adenosyl-L-methionine-dependent methyltransferases superfamily protein | 350 | no |
| 19871 | Vv08s0007g04550.t01 | AT5G03555.1 | permease, cytosine/purines, uracil, thiamine, allantoin family protein | 511 | no |
| 19921 | Vv01s0011g01260.t01 | AT2G34980.1 | phosphatidylinositolglycan synthase family protein | 303 | yes |
| 20020 | Vv00s0434g00060.t01 | AT4G35870.1 | early-responsive to dehydration stress protein (ERD4) | 850 | no |
| 20035 | Vv19s0090g00470.t01 | AT4G26965.1 | NADH:ubiquinone oxidoreductase, 17.2kDa subunit | 173 | yes |
| 20036 | Vv12s0028g01320.t01 | AT2G42780.1 | unknown protein; | 254 | yes |
| 20039 | Vv06s0004g02730.t01 | AT1G08030.1 | tyrosylprotein sulfotransferase | 512 | yes |
| 20071 | Vv10s0003g04360.t01 | AT4G05420.1 | damaged DNA binding protein 1A | 1089 | no |
| 20080 | Vv16s0098g01880.t01 | AT5G24260.1 | prolyl oligopeptidase family protein | 754 | yes |
| 20083 | Vv06s0004g01070.t01 | AT2G20480.1 | unknown protein; | 65 | yes |
| 20106 | Vv08s0040g00270.t01 | AT5G64270.1 | splicing factor, putative | 1271 | yes |
| 20151 | Vv18s0001g07100.t01 | AT5G49970.1 | pyridoxin (pyrodoxamine) 5'-phosphate oxidase | 523 | no |
| 20155 | Vv12s0057g01370.t01 | AT3G07640.1 | unknown protein; | 226 | yes |
| 20158 | Vv06s0009g00900.t01 | AT5G22040.1 | unknown protein; | 271 | yes |
| 20162 | Vv11s0103g00230.t01 | AT1G17690.1 | Digestive organ expansion factor, predicted (InterPro:IPR010678); | 753 | yes |
| 20227 | Vv14s0066g00610.t01 | NA | NA | 4565 | no |
| *20237 | Vv19s0014g01300.t01 | AT3G14900.1 | INVOLVED IN: embryo development; LOCATED IN: chloroplast; | 533 | yes |
| 20311 | Vv00s0483g00020.t01 | AT5G65490.1 | LOCATED IN: chloroplast; SGT1 (InterPro:IPR010770); | 582 | no |
| 20387 | Vv17s0000g01420.t01 | AT4G24880.1 | unknown protein; | 419 | no |
| 20537 | Vv15s0048g00210.t01 | AT3G60590.3 | unknown protein | 433 | yes |
| 20559 | Vv07s0031g02780.t01 | AT5G08720.1 | Streptomyces cyclase/dehydrase (InterPro:IPR005031);Polyketide cyclase / dehydrase and lipid transport protein | 735 | yes |
| 20676 | Vv05s0020g02950.t01 | AT5G19300.1 | Nucleic acid-binding, OB-fold-like (InterPro:IPR016027), Protein of unknown function DUF171 (InterPro:IPR003750) | 363 | yes |
| 20683 | Vv01s0137g00760.t01 | AT1G67840.1 | chloroplast sensor kinase | 627 | no |
| 20729 | Vv10s0003g03110.t01 | AT4G18260.1 | Cytochrome b561/ferric reductase transmembrane protein family | 216 | yes |
| 20730 | Vv08s0058g00890.t01 | AT3G08850.1 | HEAT repeat ;WD domain, G-beta repeat protein protein | 1363 | no |
| 20747 | Vv06s0004g03360.t01 | AT1G29900.1 | carbamoyl phosphate synthetase B | 1188 | no |
| 20795 | Vv14s0066g01700.t01 | AT5G40190.1 | RNA ligase/cyclic nucleotide phosphodiesterase family protein | 257 | yes |
| 20796 | Vv07s0005g01290.t01 | AT4G02100.1 | Heat shock protein DnaJ with tetratricopeptide repeat | 558 | yes |
| 20798 | Vv07s0005g04270.t01 | AT4G21865.1 | unknown protein; | 230 | yes |
| 20819 | Vv15s0046g01080.t01 | AT4G01040.1 | Glycosyl hydrolase superfamily protein | 435 | yes |
| 20863 | Vv16s0098g00690.t01 | AT1G18000.1 | Major facilitator superfamily protein | 472 | yes |
| 20887 | Vv14s0066g01200.t01 | AT3G01510.1 | like SEX4 1 | 583 | no |
| 20895 | Vv04s0044g00170.t01 | AT4G35520.1 | MUTL protein homolog 3 | 1220 | no |
| 20926 | Vv07s0005g06600.t01 | AT5G14105.1 | unknown protein; | 76 | yes |
| 20990 | Vv00s0179g00210.t01 | AT1G03060.1 | Beige/BEACH domain ;WD domain, G-beta repeat protein | 3534 | no |
| 21069 | Vv08s0007g03300.t01 | AT5G03900.2 | Iron-sulphur cluster biosynthesis family protein | 508 | yes |
| 21084 | Vv12s0057g00590.t01 | AT2G05170.1 | vacuolar protein sorting 11 | 961 | yes |
| 21139 | Vv14s0171g00280.t01 | AT3G01780.1 | ARM repeat superfamily protein | 1179 | yes |
| 21153 | Vv03s0038g02530.t01 | AT2G16860.1 | GCIP-interacting family protein | 340 | yes |
| 21165 | Vv17s0000g06890.t01 | NA | NA | 1641 | no |
| 21225 | Vv13s0019g04720.t01 | AT3G54860.2 | Sec1/munc18-like (SM) proteins superfamily | 597 | yes |
| 21389 | Vv14s0108g00040.t01 | AT3G28730.1 | high mobility group | 644 | yes |
| 21418 | Vv13s0067g03300.t01 | AT5G13520.1 | peptidase M1 family protein | 611 | yes |
| 21420 | Vv03s0063g00370.t01 | AT2G15620.1 | nitrite reductase 1 | 595 | no |
| 21421 | Vv06s0004g02330.t01 | AT5G04590.1 | sulfite reductase | 687 | yes |
| 21427 | Vv06s0004g02100.t01 | AT1G20050.1 | C-8,7 sterol isomerase | 237 | no |
| 21511 | Vv11s0016g02130.t01 | AT5G56900.2 | CwfJ-like family protein / zinc finger (CCCH-type) family protein | 593 | no |
| 21550 | Vv19s0027g00340.t01 | AT1G56020.1 | unknown protein; | 417 | no |
| 21551 | Vv19s0015g01940.t01 | AT5G56290.1 | peroxin 5 | 736 | yes |
| 21574 | Vv18s0001g11700.t01 | AT1G47550.1 | exocyst complex component sec3A | 886 | no |
| 21599 | Vv02s0012g02700.t01 | AT5G45610.1 | protein dimerizations | 208 | no |
| 21616 | Vv07s0005g05000.t01 | AT2G20210.1 | RNI-like superfamily protein | 245 | no |
| 21626 | Vv15s0048g02140.t01 | AT4G10180.1 | light-mediated development protein 1 / deetiolated1 (DET1) | 531 | no |
| 21634 | Vv05s0049g02320.t01 | AT2G33255.1 | Haloacid dehalogenase-like hydrolase (HAD) superfamily protein | 132 | no |
| 21658 | Vv15s0046g00300.t01 | AT2G46520.1 | cellular apoptosis susceptibility protein, putative / importin-alpha re-exporter, putative | 979 | no |
| 21721 | Vv01s0011g05690.t01 | AT1G24610.1 | Rubisco methyltransferase family protein | 483 | no |
| 21737 | Vv13s0064g00050.t01 | AT3G24090.1 | glutamine-fructose-6-phosphate transaminase (isomerizing)s;sugar binding;transaminases | 684 | yes |
| 21777 | Vv13s0019g04740.t01 | AT2G39140.1 | pseudouridine synthase family protein | 393 | yes |
| 21812 | Vv06s0004g03800.t01 | AT3G45830.1 | unknown protein; | 1392 | no |
| 21829 | Vv18s0001g04520.t01 | NA | NA | 286 | no |
| 21892 | Vv12s0028g00570.t01 | AT2G38020.1 | vacuoleless1 (VCL1) | 838 | no |
| 21909 | Vv11s0016g02620.t01 | AT5G56740.1 | histone acetyltransferase of the GNAT family 2 | 462 | no |
| 21929 | Vv01s0011g05860.t01 | AT1G14810.1 | semialdehyde dehydrogenase family protein | 379 | yes |
| 21953 | Vv11s0052g00300.t01 | AT5G57120.1 | unknown protein; | 453 | yes |
| 21999 | Vv17s0000g01170.t01 | AT4G24930.1 | thylakoid lumenal 17.9 kDa protein, chloroplast | 226 | no |
| 22009 | Vv06s0009g03810.t01 | AT2G13540.1 | ARM repeat superfamily protein | 865 | no |
| 22066 | Vv17s0000g01950.t01 | AT3G17810.1 | pyrimidine 1 | 421 | yes |
| 22075 | Vv13s0101g00510.t01 | AT2G25100.1 | Polynucleotidyl transferase, ribonuclease H-like superfamily protein | 247 | no |
| 22151 | Vv16s0022g01260.t01 | AT5G51540.1 | Zincin-like metalloproteases family protein | 531 | no |
| 22189 | Vv18s0001g13540.t01 | AT1G75550.1 | glycine-rich protein | 199 | no |
| 22239 | Vv15s0046g02880.t01 | AT1G01725.1 | unknown protein; | 80 | yes |
| 22274 | Vv06s0004g05150.t01 | AT3G46740.1 | translocon at the outer envelope membrane of chloroplasts 75-III | 808 | yes |
| 22299 | Vv03s0063g00290.t01 | AT4G34030.1 | 3-methylcrotonyl-CoA carboxylase | 570 | yes |
| 22379 | Vv19s0085g00720.t01 | AT1G16340.1 | Aldolase superfamily protein | 290 | yes |
| 22380 | Vv02s0025g02430.t01 | AT4G10750.1 | Phosphoenolpyruvate carboxylase family protein | 359 | yes |
| 22396 | Vv06s0004g00260.t01 | AT1G55480.1 | protein containing PDZ domain, a K-box domain, and a TPR region | 212 | yes |
| 22448 | Vv16s0050g01430.t01 | AT5G52810.1 | NAD(P)-binding Rossmann-fold superfamily protein | 340 | yes |
| 22460 | Vv09s0002g03770.t01 | AT3G16310.1 | mitotic phosphoprotein N' end (MPPN) family protein | 317 | yes |
| 22492 | Vv08s0058g01420.t01 | AT5G01910.1 | unknown protein; | 239 | no |
| 22530 | Vv05s0102g00720.t01 | AT3G59490.2 | unknown protein; | 329 | no |
| 22546 | Vv01s0011g05730.t01 | AT1G14850.1 | nucleoporin 155 | 1496 | no |
| 22547 | Vv05s0029g00290.t01 | AT2G31970.1 | DNA repair-recombination protein (RAD50) | 347 | no |
| 22577 | Vv11s0016g03290.t01 | AT1G77180.1 | chromatin protein family | 602 | no |
| 22626 | Vv12s0028g03140.t01 | AT4G28450.1 | nucleotide binding;protein binding | 452 | yes |
| 22652 | Vv04s0008g05610.t01 | AT4G32190.1 | Myosin heavy chain-related protein | 774 | yes |
| 22665 | Vv08s0040g02260.t01 | AT5G03110.1 | LOCATED IN: plasma membrane; protamine P1 family protein | 468 | no |
| 22721 | Vv15s0048g00430.t01 | AT1G02020.1 | nitroreductase family protein | 586 | no |
| 22765 | Vv02s0012g00180.t01 | NA | Endoribonuclease XendoU (IPR018998) | 444 | yes |
| 22784 | Vv00s0684g00040.t01 | AT4G35760.1 | NAD(P)H dehydrogenase (quinone)s | 290 | no |
| 22788 | Vv18s0072g00720.t01 | AT1G76850.1 | exocyst complex component sec5 | 978 | yes |
| *22797 | Vv15s0046g02330.t01 | AT2G45990.1 | unknown protein; LOCATED IN: chloroplast, chloroplast stroma; | 268 | yes |
| 22845 | Vv18s0075g00120.t01 | AT1G71990.1 | fucosyltransferase 13 | 403 | no |
| 22885 | Vv03s0088g00320.t01 | AT1G17870.1 | ethylene-dependent gravitropism-deficient and yellow-green-like 3 | 456 | yes |
| 22886 | Vv04s0008g07000.t01 | AT2G24830.1 | zinc finger (CCCH-type) family protein / D111/G-patch domain-containing protein | 496 | yes |
| *22896 | Vv06s0004g08380.t01 | AT5G58250.1 | unknown protein; LOCATED IN: thylakoid, chloroplast; | 227 | no |
| 22897 | Vv05s0102g00430.t01 | AT3G19810.1 | Protein of unknown function (DUF177) | 320 | yes |
| 22919 | Vv19s0014g01810.t01 | AT1G53200.1 | unknown protein; | 580 | no |
| **22999 | Vv06s0004g07560.t01 | AT1G16870.1 | mitochondrial 28S ribosomal protein S29-related | 457 | yes |
| 23084 | Vv03s0063g02450.t01 | AT4G34450.1 | coatomer gamma-2 subunit, putative / gamma-2 coat protein, putative / gamma-2 COP, putative | 887 | no |
| 23122 | Vv05s0102g00250.t01 | AT1G05950.1 | unknown protein; | 546 | no |
| 23157 | Vv10s0071g01040.t01 | AT5G63670.1 | SPT4 homolog 2 | 114 | yes |
| 23194 | Vv00s0415g00040.t01 | AT2G22660.2 | Protein of unknown function (duplicated DUF1399) | 825 | yes |
| 23248 | Vv07s0130g00070.t01 | AT5G23630.1 | phosphate deficiency response 2 | 1190 | yes |
| 23308 | Vv02s0025g03430.t01 | AT1G63660.1 | GMP synthase (glutamine-hydrolyzing), putative / glutamine amidotransferase, putative | 534 | yes |
| 23311 | Vv02s0012g01050.t01 | AT1G32500.1 | non-intrinsic ABC protein 6 | 375 | yes |
| 23332 | Vv11s0016g05020.t01 | AT5G20170.1 | RNA polymerase II transcription mediators | 660 | no |
| 23368 | Vv14s0006g01740.t01 | AT1G66510.1 | AAR2 protein family | 394 | no |
| 23393 | Vv17s0000g03970.t01 | AT3G18480.1 | CCAAT-displacement protein alternatively spliced product | 671 | yes |
| 23394 | Vv11s0065g00450.t01 | AT2G26590.1 | regulatory particle non-ATPase 13 | 320 | yes |
| 23395 | Vv18s0001g03600.t01 | AT3G02760.1 | Class II aaRS and biotin synthetases superfamily protein | 607 | yes |
| 23406 | Vv00s0227g00070.t01 | AT5G57700.3 | BNR/Asp-box repeat family protein | 151 | yes |
| 23408 | Vv10s0092g00350.t01 | AT4G19490.2 | VPS54 | 1041 | no |
| 23444 | Vv17s0000g01530.t01 | AT5G63440.2 | Protein of unknown function (DUF167) | 232 | no |
| *23490 | Vv08s0058g00370.t01 | AT5G01590.1 | unknown protein; LOCATED IN: chloroplast, chloroplast envelope; | 506 | yes |
| 23532 | Vv06s0004g02820.t01 | AT2G28390.1 | SAND family protein | 615 | yes |
| 23551 | Vv01s0011g00850.t01 | AT2G03420.1 | unknown protein; | 177 | no |
| 23559 | Vv04s0023g00750.t01 | AT3G24080.1 | KRR1 family protein | 637 | yes |
| 23583 | Vv18s0001g03250.t01 | AT1G44835.2 | YbaK/aminoacyl-tRNA synthetase-associated domain | 320 | yes |
| 23588 | Vv01s0127g00060.t01 | AT1G10760.1 | Pyruvate phosphate dikinase, PEP/pyruvate binding domain | 1470 | no |
| 23591 | Vv18s0001g04980.t01 | AT1G36160.1 | acetyl-CoA carboxylase 1 | 2258 | yes |
| 23653 | Vv17s0000g05140.t01 | AT1G18600.1 | RHOMBOID-like protein 12 | 333 | no |
| *23695 | Vv00s0333g00020.t01 | AT5G48470.1 | unknown protein; LOCATED IN: chloroplast; | 338 | no |
| 23770 | Vv14s0128g00470.t01 | AT5G27560.1 | Domain of unknown function (DUF1995) | 353 | yes |
| 23841 | Vv07s0130g00050.t01 | AT1G47570.1 | RING/U-box superfamily protein | 462 | yes |
| 23969 | Vv04s0008g02480.t01 | AT4G31430.2 | unknown protein; LOCATED IN: plasma membrane; | 627 | yes |
| 24003 | Vv16s0039g00080.t01 | AT1G17820.1 | Putative integral membrane protein conserved region (DUF2404) | 457 | yes |
| 24016 | Vv06s0004g07730.t01 | AT1G06950.1 | translocon at the inner envelope membrane of chloroplasts 110 | 1007 | yes |
| 24027 | Vv17s0000g04050.t01 | AT1G73920.1 | alpha/beta-Hydrolases superfamily protein | 691 | yes |
| 24034 | Vv03s0063g02030.t01 | AT4G34350.1 | 4-hydroxy-3-methylbut-2-enyl diphosphate reductase | 465 | yes |
| 24049 | Vv13s0064g01450.t01 | AT5G42850.1 | Thioredoxin superfamily protein | 132 | yes |
| 24067 | Vv17s0000g04890.t01 | AT2G03800.1 | D-aminoacyl-tRNA deacylases | 316 | no |
| 24076 | Vv05s0020g02800.t01 | AT5G19150.1 | pfkB-like carbohydrate kinase family protein | 354 | yes |
| 24102 | Vv19s0015g01400.t01 | AT3G13235.1 | ubiquitin family protein | 410 | no |
| 24138 | Vv05s0029g00640.t01 | AT3G23910.1 | RNA-directed DNA polymerase (reverse transcriptase)-related family protein; | 425 | no |
| 24148 | Vv18s0001g01650.t01 | AT3G07730.1 | unknown protein; | 421 | no |
| *24157 | Vv17s0000g00930.t01 | AT1G73470.1 | unknown protein; LOCATED IN: chloroplast; | 354 | no |
| 24173 | Vv04s0008g02950.t01 | AT4G31210.1 | DNA topoisomerase, type IA, core | 913 | no |
| 24310 | Vv07s0005g02360.t01 | AT1G02330.1 | Hepatocellular carcinoma-associated antigen 59 (InterPro:IPR010756); | 296 | yes |
| 24334 | Vv17s0119g00350.t01 | AT1G48430.1 | Dihydroxyacetone kinase | 594 | yes |
| *24385 | Vv00s0201g00060.t01 | AT5G50350.1 | unknown protein; INVOLVED IN: response to oxidative stress; LOCATED IN: chloroplast; | 572 | no |
| 24388 | Vv14s0006g00650.t01 | AT2G18900.1 | Transducin/WD40 repeat-like superfamily protein | 294 | yes |
| 24399 | Vv02s0087g00160.t01 | NA | Uncharacterised protein family SERF (IPR007513); Zinc finger, C2H2-like (IPR015880) | 68 | yes |
| 24405 | Vv02s0012g01840.t01 | AT5G46850.1 | unknown protein; | 329 | yes |
| 24459 | Vv08s0007g06710.t01 | AT3G12050.1 | Aha1 domain-containing protein | 347 | yes |
| 24511 | Vv18s0157g00050.t01 | AT1G71696.2 | carboxypeptidase D, putative | 493 | no |
| 24560 | Vv01s0011g00480.t01 | AT1G26640.1 | Amino acid kinase family protein | 340 | yes |
| 24589 | Vv08s0056g01250.t01 | AT3G09360.1 | Cyclin/Brf1-like TBP-binding protein | 622 | yes |
| 24682 | Vv17s0000g03250.t01 | AT3G18240.1 | Ribosomal protein S24/S35, mitochondrial | 420 | yes |
| 24891 | Vv18s0001g14380.t01 | AT5G42470.1 | Brain/reproductive organ-expressed protein (InterPro:IPR010358); | 375 | no |
| 24907 | Vv06s0004g01860.t01 | AT3G20870.1 | ZIP metal ion transporter family | 276 | yes |
| 24940 | Vv01s0146g00420.t01 | NA | Endonuclease I (IPR007346); | 99 | yes |
| 24945 | Vv17s0000g00660.t01 | AT3G30841.1 | Cofactor-independent phosphoglycerate mutase | 492 | yes |
| 24950 | Vv14s0128g00710.t01 | AT1G09010.1 | glycoside hydrolase family 2 protein | 973 | yes |
| 24954 | Vv03s0063g01360.t01 | AT2G15860.2 | unknown protein; | 510 | yes |
| 24973 | Vv01s0011g01090.t01 | AT5G44710.1 | Ribosomal protein S27/S33, mitochondrial (InterPro:IPR013219); | 102 | yes |
| 24995 | Vv18s0001g09310.t01 | AT5G43130.1 | TBP-associated factor 4 | 926 | yes |
| 25032 | Vv14s0066g00990.t01 | AT5G14600.1 | S-adenosyl-L-methionine-dependent methyltransferases superfamily protein | 349 | yes |
| 25056 | Vv08s0032g00330.t01 | AT1G80770.1 | P-loop containing nucleoside triphosphate hydrolases superfamily protein | 353 | yes |
| 25075 | Vv12s0142g00260.t01 | AT3G58800.1 | unknown protein; | 346 | no |
| 25091 | Vv08s0007g08940.t01 | AT2G41620.1 | Nucleoporin interacting component (Nup93/Nic96-like) family protein | 863 | no |
| 25094 | Vv04s0008g04310.t01 | AT5G11450.1 | Mog1/PsbP/DUF1795-like photosystem II reaction center PsbP family protein | 284 | yes |
| 25134 | Vv06s0004g06690.t01 | AT1G07210.1 | Ribosomal protein S18 | 322 | yes |
| 25153 | Vv18s0001g02070.t01 | AT1G71780.1 | unknown protein; | 186 | yes |
| 25159 | Vv14s0128g00150.t01 | AT3G05210.1 | nucleotide repair protein, putative | 398 | no |
| 25191 | Vv18s0166g00300.t01 | AT5G63890.2 | histidinol dehydrogenase | 562 | yes |
| 25294 | Vv01s0011g04400.t01 | AT2G01120.1 | origin recognition complex subunit 4 | 456 | no |
| 25321 | Vv11s0052g01130.t01 | AT5G09390.1 | CD2-binding protein-related | 419 | yes |
| 25343 | Vv06s0009g02170.t01 | AT5G12290.1 | dgd1 suppressor 1 | 518 | no |
| 25367 | Vv09s0002g05110.t01 | AT1G80410.1 | tetratricopeptide repeat (TPR)-containing protein | 1332 | no |
| 25373 | Vv11s0016g05610.t01 | AT4G11120.1 | translation elongation factor Ts (EF-Ts), putative | 378 | yes |
| 25388 | Vv03s0097g00590.t01 | AT3G20000.1 | translocase of the outer mitochondrial membrane 40 | 309 | yes |
| 25419 | Vv13s0067g02890.t01 | AT4G19160.2 | unknown protein; | 423 | no |
| 25431 | Vv18s0001g09200.t01 | AT1G27750.1 | nucleic acid binding | 1331 | no |
| 25437 | Vv11s0016g02990.t01 | AT4G26300.1 | Arginyl-tRNA synthetase, class Ic | 637 | no |
| 25442 | Vv14s0066g00490.t01 | AT5G40480.1 | embryo defective 3012 | 874 | no |
| 25443 | Vv10s0042g00140.t01 | AT5G23300.1 | pyrimidine d | 445 | no |
| 25531 | Vv01s0011g03120.t01 | AT1G29700.1 | Metallo-hydrolase/oxidoreductase superfamily protein | 340 | yes |
| *25542 | Vv18s0001g08630.t01 | AT1G44920.1 | unknown protein; LOCATED IN: chloroplast; | 260 | yes |
| 25562 | Vv10s0116g00770.t01 | AT1G05205.1 | unknown protein; | 86 | yes |
| 25563 | Vv00s0591g00010.t01 | AT5G41150.1 | Restriction endonuclease, type II-like superfamily protein | 1324 | no |
| 25572 | Vv13s0158g00140.t01 | AT3G63460.1 | transducin family protein / WD-40 repeat family protein | 1099 | yes |
| 25574 | Vv16s0100g01250.t01 | AT5G52220.1 | CONTAINS InterPro DOMAIN/s: Chromosome transmission fidelity protein 8 (InterPro:IPR018607); | 140 | yes |
| 25600 | Vv18s0001g02950.t01 | AT1G48200.1 | unknown protein; LOCATED IN: endomembrane system; | 112 | yes |
| 25610 | Vv04s0008g01350.t01 | AT5G11980.1 | conserved oligomeric Golgi complex component-related / COG complex component-related | 571 | yes |
| 25642 | Vv04s0008g02440.t01 | AT5G10710.2 | unknown protein; | 326 | no |
| 25671 | Vv02s0025g00400.t01 | AT1G63980.1 | D111/G-patch domain-containing protein | 370 | yes |
| 25674 | Vv08s0007g04460.t01 | AT5G03560.2 | Tetratricopeptide repeat (TPR)-like superfamily protein | 193 | yes |
| 25695 | Vv12s0035g01810.t01 | AT5G49880.1 | mitotic checkpoint family protein | 717 | yes |
| 25706 | Vv00s0586g00020.t01 | AT2G47960.1 | unknown protein; Protein of unknown function DUF974 (InterPro:IPR010378); | 438 | no |
| 25726 | Vv18s0122g01370.t01 | AT1G42440.1 | unknown protein; | 801 | yes |
| 25749 | Vv06s0061g01260.t01 | AT5G22640.1 | MORN (Membrane Occupation and Recognition Nexus) repeat-containing protein | 859 | yes |
| 25773 | Vv03s0038g00880.t01 | AT2G16570.1 | GLN phosphoribosyl pyrophosphate amidotransferase 1 | 582 | yes |
| 25786 | Vv09s0002g00200.t01 | AT5G50420.1 | O-fucosyltransferase family protein | 559 | yes |
| 25796 | Vv04s0043g00330.t01 | AT5G16550.1 | unknown protein; | 188 | yes |
| 25799 | Vv10s0116g00970.t01 | AT4G04350.1 | tRNA synthetase class I (I, L, M and V) family protein | 971 | no |
| 25801 | Vv07s0130g00260.t01 | AT3G56570.1 | SET domain-containing protein | 500 | no |
| 25803 | Vv08s0040g00570.t01 | AT2G41020.1 | WW domain-containing protein | 532 | no |
| 25841 | Vv03s0038g00450.t01 | AT4G34640.1 | squalene synthase 1 | 413 | yes |
| 25843 | Vv05s0029g01230.t01 | AT4G13750.1 | Histidine kinase-, DNA gyrase B-, and HSP90-like ATPase family protein | 690 | no |
| 25845 | Vv10s0003g04560.t01 | AT2G34680.1 | Outer arm dynein light chain 1 protein | 1717 | no |
| 25871 | Vv04s0159g00020.t01 | AT3G23900.1 | RNA recognition motif (RRM)-containing protein | 859 | no |
| 25920 | Vv18s0001g02630.t01 | AT1G21760.1 | F-box protein 7 | 328 | no |
| 25932 | Vv05s0029g00110.t01 | AT4G13630.1 | Protein of unknown function, DUF593 | 797 | yes |
| 25946 | Vv11s0016g03870.t01 | AT5G19660.1 | SITE-1 protease | 1046 | no |
| 25990 | Vv03s0017g02210.t01 | AT4G15140.1 | unknown protein; | 137 | yes |
| 25994 | Vv01s0137g00440.t01 | AT1G13120.1 | embryo defective 1745 | 415 | no |
| 26008 | Vv10s0116g00450.t01 | AT5G15170.1 | tyrosyl-DNA phosphodiesterase-related | 678 | no |
| 26015 | Vv19s0085g01080.t01 | AT1G17680.1 | tetratricopeptide repeat (TPR)-containing protein | 355 | no |
| 26037 | Vv15s0046g01940.t01 | AT4G00290.1 | Mechanosensitive ion channel protein | 403 | yes |
| 26057 | Vv19s0014g03530.t01 | AT5G54290.1 | cytochrome c biogenesis protein family | 358 | yes |
| 26136 | Vv08s0032g01140.t01 | AT5G16280.1 | Tetratricopeptide repeat (TPR)-like superfamily protein | 1262 | no |
| 26172 | Vv00s0187g00350.t01 | AT4G12610.2 | transcription activators;DNA binding;RNA polymerase II transcription factors;catalytics;transcription initiation factors | 539 | no |
| 26193 | Vv08s0007g01780.t01 | AT3G09100.2 | mRNA capping enzyme family protein | 677 | no |
| 26228 | Vv00s2547g00010.t01 | NA | NA | 230 | yes |
| 26230 | Vv04s0044g01210.t01 | AT5G64070.1 | phosphatidylinositol 4-OH kinase beta1 | 1067 | no |
| *26323 | Vv06s0061g00330.t01 | AT3G45050.2 | unknown protein; | 160 | no |
| 26353 | Vv09s0018g01620.t01 | AT1G50500.1 | Membrane trafficking VPS53 family protein | 826 | yes |
| 26367 | Vv18s0001g08180.t01 | AT4G31770.1 | debranching enzyme 1 | 407 | no |
| 26449 | Vv14s0060g00590.t01 | AT5G26760.2 | unknown protein; Protein of unknown function DUF408 (InterPro:IPR007308); | 659 | yes |
| **26493 | Vv09s0002g08990.t01 | AT1G80700.1 | unknown protein; LOCATED IN: mitochondrion; | 165 | yes |
| 26508 | Vv11s0052g00520.t01 | NA | NA | 296 | no |
| **26554 | Vv00s2171g00010.t01 | AT5G48530.1 | unknown protein;LOCATED IN: mitochondrion; | 162 | yes |
| 26563 | Vv11s0016g00420.t01 | AT2G26460.1 | RED family protein | 566 | yes |
| 26586 | Vv13s0074g00420.t01 | AT4G40045.1 | unknown protein; | 193 | yes |
| 26601 | Vv01s0011g00230.t01 | AT4G30000.1 | Dihydropterin pyrophosphokinase / Dihydropteroate synthase | 511 | yes |
| 26663 | Vv19s0014g00980.t01 | AT1G78680.1 | gamma-glutamyl hydrolase 2 | 384 | yes |
| 26767 | Vv04s0008g06320.t01 | AT2G25280.1 | UPF0103/Mediator of ErbB2-driven cell motility (Memo), related (InterPro:IPR002737); | 291 | no |
| 26799 | Vv02s0012g01800.t01 | AT2G35500.1 | shikimate kinase like 2 | 371 | yes |
| 26811 | Vv18s0001g14460.t01 | AT4G24175.1 | unknown protein; | 295 | no |
| *26896 | Vv11s0052g00920.t01 | AT5G57460.1 | unknown protein; LOCATED IN: plasma membrane, chloroplast; | 621 | yes |
| 26900 | Vv18s0072g00410.t01 | AT1G43580.1 | Sphingomyelin synthetase family protein | 432 | yes |
| *26901 | Vv14s0081g00710.t01 | AT3G04550.1 | unknown protein; LOCATED IN: chloroplast stroma, chloroplast; | 443 | yes |
| 26941 | Vv12s0059g01290.t01 | AT2G32980.1 | unknown protein; | 300 | yes |
| *26943 | Vv00s0246g00160.t01 | ATCG01020.1 | ribosomal protein L32 | 57 | yes |
| 26944 | Vv18s0001g04540.t01 | AT4G08540.1 | DNA-directed RNA polymerase II protein | 478 | no |
| 26952 | Vv01s0026g00750.t01 | AT1G24050.1 | RNA-processing, Lsm domain | 167 | yes |
| 26972 | Vv04s0044g00140.t01 | AT2G17550.1 | unknown protein; | 902 | yes |
| 27019 | Vv07s0130g00190.t01 | AT5G23570.1 | XS domain-containing protein / XS zinc finger domain-containing protein-related | 738 | no |
| 27104 | Vv18s0001g03560.t01 | AT4G10080.1 | unknown protein; | 415 | yes |
| 27110 | Vv04s0008g06810.t01 | AT5G25060.1 | RNA recognition motif (RRM)-containing protein | 964 | yes |
| 27150 | Vv13s0019g03240.t01 | AT1G51740.1 | syntaxin of plants 81 | 314 | yes |
| 27156 | Vv18s0072g00300.t01 | AT1G76730.1 | NagB/RpiA/CoA transferase-like superfamily protein | 354 | yes |
| *27197 | Vv06s0009g03770.t01 | AT3G09150.2 | phytochromobilin:ferredoxin oxidoreductase, chloroplast / phytochromobilin synthase (HY2) | 330 | yes |
| 27236 | Vv08s0007g00060.t01 | AT5G37830.1 | oxoprolinase 1 | 1269 | no |
| *27300 | Vv11s0016g03910.t01 | AT5G19540.1 | unknown protein; LOCATED IN: chloroplast; | 444 | yes |
| 27346 | Vv07s0129g00540.t01 | AT4G37460.1 | Tetratricopeptide repeat (TPR)-like superfamily protein | 1068 | no |
| 27367 | Vv01s0011g01690.t01 | AT5G22480.1 | ZPR1 zinc-finger domain protein | 489 | yes |
| 27377 | Vv11s0016g03000.t01 | AT4G29380.1 | protein kinase family protein / WD-40 repeat family protein | 1545 | no |
| 27379 | Vv15s0107g00240.t01 | AT5G17610.1 | unknown protein; | 130 | yes |
| 27412 | Vv18s0001g09070.t01 | AT1G59600.1 | ZCW7 | 365 | no |
| 27449 | Vv04s0043g00860.t01 | AT2G38430.1 | unknown protein; | 413 | no |
| 27463 | Vv06s0004g02870.t01 | AT2G28430.1 | unknown protein; | 85 | yes |
| 27469 | Vv18s0001g09370.t01 | AT5G65860.1 | ankyrin repeat family protein | 327 | yes |
| 27537 | Vv17s0000g04070.t01 | AT3G18510.1 | unknown protein; | 63 | yes |
| 27559 | Vv12s0028g01340.t01 | AT2G30910.1 | actin-related protein C1A | 378 | yes |
| 27569 | Vv06s0004g06130.t01 | AT2G29630.2 | thiaminC | 712 | no |
| 27610 | Vv18s0001g00440.t01 | AT2G44270.1 | repressor of lrx1 | 370 | yes |
| 27635 | Vv05s0049g00190.t01 | AT5G43500.1 | actin-related protein 9 | 595 | yes |
| 27672 | Vv05s0020g04640.t01 | AT1G04230.1 | Protein of unknown function (DUF2361) | 337 | yes |
| 27717 | Vv14s0006g00380.t01 | AT3G52640.2 | Zn-dependent exopeptidases superfamily protein | 671 | yes |
| 27753 | Vv09s0002g03240.t01 | AT4G01590.1 | unknown protein; | 211 | yes |
| **27760 | Vv14s0006g01750.t01 | AT5G17460.1 | unknown protein;INVOLVED IN: response to salt stress; LOCATED IN: mitochondrion; | 312 | yes |
| *27801 | Vv18s0089g01390.t01 | AT5G44650.1 | Encodes a chloroplast protein that induces tolerance to multiple environmental stresses and reduces photooxidative damage. | 254 | yes |
| 27806 | Vv09s0002g01860.t01 | AT3G13940.1 | DNA binding;DNA-directed RNA polymerases | 421 | yes |
| 27857 | Vv16s0039g01610.t01 | AT3G42050.1 | vacuolar ATP synthase subunit H family protein | 483 | yes |
| 28066 | Vv16s0039g02650.t01 | AT2G36740.1 | sequence-specific DNA binding transcription factors;DNA binding;DNA binding | 301 | yes |
| 28167 | Vv06s0004g03970.t01 | AT3G46220.1 | unknown protein; | 820 | no |
| 28181 | Vv05s0077g02270.t01 | AT3G06868.1 | unknown protein; | 405 | yes |
| 28240 | Vv07s0031g03120.t01 | AT1G05970.2 | RNA-binding (RRM/RBD/RNP motifs) family protein | 259 | yes |
| 28981 | Vv05s0020g03160.t01 | AT1G05360.1 | SNARE associated Golgi protein family | 439 | no |
