# Supplementary material for: Transcriptome Sequences Resolve Deep Relationships of the Grape Family
Source: PLoS One. 2013 Sep 17;8(9):e74394. doi: 10.1371/journal.pone.0074394 (PMC3775763; doi:10.1371/journal.pone.0074394)
Supplement: Table S4 — Topological stability as estimated by bootstrap support of nodes with the maximum likelihood method by randomly reducing the gene number by 10, starting from the 229 gene data set. (DOCX) [file pone.0074394.s008.docx]

**Table S4.**  Topological stability as estimated by bootstrap support of nodes with the maximum likelihood method by randomly reducing the gene number by 10, starting from the 229 gene data set. Node numbers N1-N12 are shown in Fig. 2.

| **#Gene** | **N1** | **N2** | **N3** | **N4** | **N5** | **N6** | **N7** | **N8** | **N9** | **N10** | **N11** | **N12** |
| --- | --- | --- | --- | --- | --- | --- | --- | --- | --- | --- | --- | --- |
| 10 | 100 | 100 | 100 | 98.27 | 92.5 | 100 | 87.57 | 99.93 | 100 | 100 | 100 | 100 |
| 20 | 100 | 100 | 100 | 99.57 | 94.53 | 100 | 98.53 | 100 | 100 | 100 | 100 | 100 |
| 30 | 100 | 100 | 100 | 99.97 | 98.4 | 100 | 98.8 | 100 | 100 | 100 | 100 | 100 |
| 40 | 100 | 100 | 100 | 100 | 99.63 | 100 | 99.87 | 100 | 100 | 100 | 100 | 100 |
| 50 | 100 | 100 | 100 | 100 | 99.97 | 100 | 99.6 | 100 | 100 | 100 | 100 | 100 |
| 60 | 100 | 100 | 100 | 100 | 100 | 100 | 99.9 | 100 | 100 | 100 | 100 | 100 |
| 70 | 100 | 100 | 100 | 100 | 99.87 | 100 | 99.97 | 100 | 100 | 100 | 100 | 100 |
| 80 | 100 | 100 | 100 | 100 | 99.97 | 100 | 100 | 100 | 100 | 100 | 100 | 100 |
| 90 | 100 | 100 | 100 | 100 | 100 | 100 | 100 | 100 | 100 | 100 | 100 | 100 |
| 100 | 100 | 100 | 100 | 100 | 100 | 100 | 100 | 100 | 100 | 100 | 100 | 100 |
| 110 | 100 | 100 | 100 | 100 | 100 | 100 | 100 | 100 | 100 | 100 | 100 | 100 |
| 120 | 100 | 100 | 100 | 100 | 100 | 100 | 100 | 100 | 100 | 100 | 100 | 100 |
| 130 | 100 | 100 | 100 | 100 | 100 | 100 | 100 | 100 | 100 | 100 | 100 | 100 |
| 140 | 100 | 100 | 100 | 100 | 100 | 100 | 100 | 100 | 100 | 100 | 100 | 100 |
| 150 | 100 | 100 | 100 | 100 | 100 | 100 | 100 | 100 | 100 | 100 | 100 | 100 |
| 160 | 100 | 100 | 100 | 100 | 100 | 100 | 100 | 100 | 100 | 100 | 100 | 100 |
| 170 | 100 | 100 | 100 | 100 | 100 | 100 | 100 | 100 | 100 | 100 | 100 | 100 |
| 180 | 100 | 100 | 100 | 100 | 100 | 100 | 100 | 100 | 100 | 100 | 100 | 100 |
| 190 | 100 | 100 | 100 | 100 | 100 | 100 | 100 | 100 | 100 | 100 | 100 | 100 |
| 200 | 100 | 100 | 100 | 100 | 100 | 100 | 100 | 100 | 100 | 100 | 100 | 100 |
| 210 | 100 | 100 | 100 | 100 | 100 | 100 | 100 | 100 | 100 | 100 | 100 | 100 |
| 220 | 100 | 100 | 100 | 100 | 100 | 100 | 100 | 100 | 100 | 100 | 100 | 100 |
